# Eltrombopag directly inhibits BAX and prevents cell death

**DOI:** 10.1038/s41467-021-21224-1

**Published:** 2021-02-18

**Authors:** Adam Z. Spitz, Emmanouil Zacharioudakis, Denis E. Reyna, Thomas P. Garner, Evripidis Gavathiotis

**Affiliations:** 1 Department of Biochemistry, Albert Einstein College of Medicine, Bronx, NY USA; 2 Department of Medicine, Albert Einstein College of Medicine, Bronx, NY USA; 3 Albert Einstein Cancer Center, Albert Einstein College of Medicine, Bronx, NY USA; 4 Wilf Family Cardiovascular Research Institute, Albert Einstein College of Medicine, Bronx, NY USA; 5 Institute of Aging Research, Albert Einstein College of Medicine, Bronx, NY USA

**Keywords:** Structural biology, Apoptosis, Target validation, Target identification

## Abstract

The BCL-2 family protein BAX has essential activity in mitochondrial regulation of cell death. While BAX activity ensures tissue homeostasis, when dysregulated it contributes to aberrant cell death in several diseases. During cellular stress BAX is transformed from an inactive cytosolic conformation to a toxic mitochondrial oligomer. Although the BAX transformation process is not well understood, drugs that interfere with this process are useful research tools and potential therapeutics. Here, we show that Eltrombopag,  an FDA-approved drug,  is a direct inhibitor of BAX. Eltrombopag binds the BAX trigger site distinctly from BAX activators, preventing them from triggering BAX conformational transformation and simultaneously promoting stabilization of the inactive BAX structure. Accordingly, Eltrombopag is capable of inhibiting BAX-mediated apoptosis induced by cytotoxic stimuli. Our data demonstrate structure-function insights into a mechanism of BAX inhibition and reveal a mechanism for Eltrombopag that may expand its use in diseases of uncontrolled cell death.

## Abstract (web summary)

The BCL-2 family protein BAX functions to regulate mitochondria-driven cell death. Here the authors show that the drug Eltrombopag inhibits BAX and prevents apoptosis induced by cytotoxic stimuli.

## Introduction

BCL-2 family proteins are principal regulators of apoptosis in health and diseases^1–4^. Mitochondrial outer membrane permeabilization (MOMP) is a key event that defines apoptotic cell death^5,6^. MOMP releases apoptogenic factors such as cytochrome *c* into the cytosol, which in turn irreversibly execute the apoptotic signaling cascade^5,6^. Pro-apoptotic BCL-2 proteins BAX and BAK play a key role in this process due to their ability to transform into mitochondrial outer membrane-embedded oligomers that induce MOMP^7,8^. In cells, BAX and BAK can exist as an inactive monomer, autoinhibited homodimer, or a neutralized conformation bound to anti-apoptotic BCL-2 family members such as BCL-2, BCL-xL, and MCL-1^9–13^. The pro-apoptotic “BH3-only” proteins such as BIM, BID, and PUMA, which comprise the third class of the BCL-2 family, sense cellular stress and utilize their BCL-2 homology 3 (BH3) domain helix to either neutralize the anti-apoptotic BCL-2 proteins and/or directly activate pro-apoptotic BAX and BAK and initiate their conformational transformation^13–15^.

**Fig. 1:**
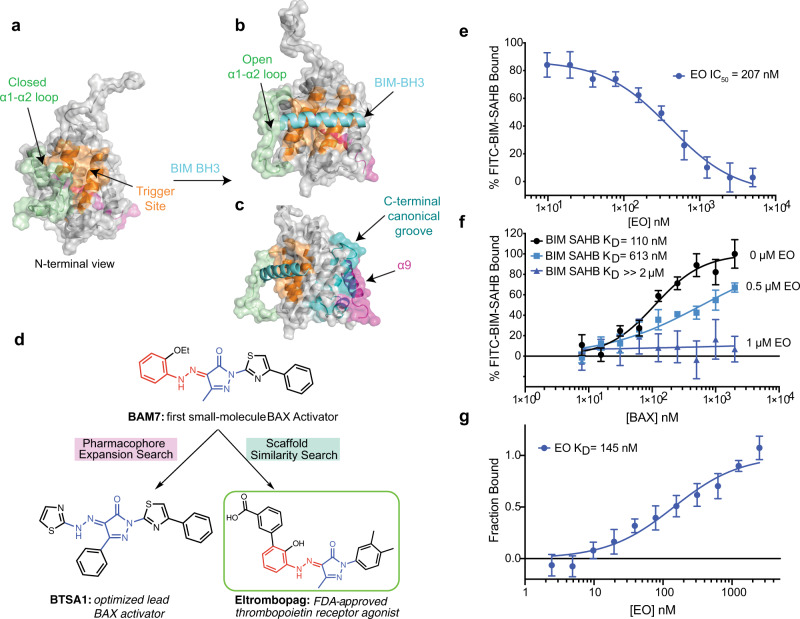
Eltrombopag potently binds BAX. **a** Surface representation of the inactive BAX structure (PDB 1F16: 10.2210/pdb1F16/pdb) showing the location of the BAX trigger site (orange) and closed α1–α2 loop (green). **b**, **c** Surface representation of the BIM–BH3-bound active conformation (PDB 2K7W: 10.2210/pdb2K7W/pdb) showing binding of BIM–BH3 (cyan) with opened α1-α2 loop (green) at the trigger site (**b**) and the location of α9 bound to the C-terminal canonical groove (**c**). **d** Chemical structures of BAM7, BTSA1, and EO derived by similarity search. The 3-methyl pyrazolone (blue) and phenylhydrazone (red) groups are highlighted for clarity. **e** Competitive fluorescence polarization (FP) binding assay of EO. Data are representative of three independent experiments, each *n* = 3 ± SEM. **f** Binding affinity of FITC-BIM–SAHB to BAX by FP in the presence of EO. Data represent *n* = 2 ± SEM from two independent experiments. **g** Microscale thermophoresis direct binding of EO to BAX-4C. Data are representative of three independent experiments each *n* = 3 ± SEM. Source data for this figure is provided.

BAX activation is a dynamic process that occurs upon binding of a BH3-only protein with its BH3 domain helix to the N-terminal BAX trigger site (α1, α6 helices), inducing several conformational changes^16–22^ (Fig. 1a, b). The release of the helix α9 from the C-terminal canonical site of BAX, formed by α3-α5 helices, allows BAX to translocate from the cytosol and insert into the mitochondrial outer membrane (MOM) (Fig. 1c)^18–21^. Once translocated, BAX forms homo-oligomeric pores which permeabilize the MOM^20–22^.

While BAX-mediated cell death contributes to tissue homeostasis and killing of malfunctioning cells, genetic deletion of BAX alone has shown successful protection from excess cell death in various disease models^23–27^. Small molecules that can directly modulate BAX can be useful probes to investigate the role of BAX in the context of various biological mechanisms and disease models^28,29^. Such chemical probes can aid in identifying BAX regulatory sites and elucidate the complex conformational transformation of BAX. Indeed, we previously identified small-molecule BAX inhibitors that bind an allosteric binding site on inactive BAX, which is distinct from previously known regulatory sites^30^. These small molecules trap BAX in an inactive state and have shown efficacy in preventing cardiomyocyte cell death in pre-clinical animal models of doxorubicin-induced cardiomyopathy^31^.

The discovery of novel BAX targeting small molecules offers an opportunity to develop them into drugs. However, repurposing of clinically approved small molecules is a highly attractive goal given the tremendous challenges of de-novo drug discovery and development^32^. While searching for direct BAX modulators based on the previous small-molecule BAX activators^33,34^, we identified Eltrombopag (EO), an FDA-approved thrombopoietin receptor agonist and iron chelator that is used to increase blood platelet counts due to chronic immune thrombocytopenia^35,36^. Despite some similarity with the BAX activators, EO proved instead to be a direct inhibitor of BAX. Thus, we characterized the mechanism of BAX inhibition providing structural and functional insights and investigated whether EO can target BAX intracellularly and inhibit BAX-mediated cell death.

## Results

### Eltrombopag binds to BAX

To identify small-molecule BAX modulators, we explored pharmacophore enhancement and substructure similarity computational methods based on the small-molecule activator of BAX, BAM7; **1**^33^. Searching a library of FDA-approved small molecules using the 3-methyl pyrazolone and phenylhydrazone core as a query, we identified as a hit, Eltrombopag (EO; **2**) (Fig. 1d). EO bears a striking similarity to BAM7, sharing the 3-methyl pyrazolone and phenylhydrazone core, however, substitutions at either side to this core with dimethylphenyl and benzoic acid markedly distinguish EO from BAM7 and the optimized lead BAX activator, BTSA1, **3**^33,34^ (Fig. 1d).

We hypothesized EO would exhibit some binding interaction with the N-terminal BAX trigger site. We therefore used a competitive fluorescence polarization assay (FPA)^33^ based on the interaction between recombinant BAX and fluorescently-labeled stapled BIM–BH3 peptide (FITC-BIM–SAHB)^17^. EO dose-dependently competed FITC-BIM–SAHB with a remarkable half-maximal inhibition IC_50_ of 207 nM (Fig. 1e). Titrations of BAX to a constant concentration FITC-BIM–SAHB exhibited a decreased affinity in the presence of constant concentrations of EO (Fig. 1f). Direct binding of EO to BAX was demonstrated using microscale thermophoresis (MST) with a calculated dissociation constant K_D_ of 143 nM (Fig. 1g and Supplementary Fig. 1a, b). The IC_50_ and K_D_ are consistent with a competitive binding mechanism where EO directly displaces FITC-BIM–SAHB from the N-terminal trigger site of BAX.

### Eltrombopag inhibits BAX activation

**Fig. 2:**
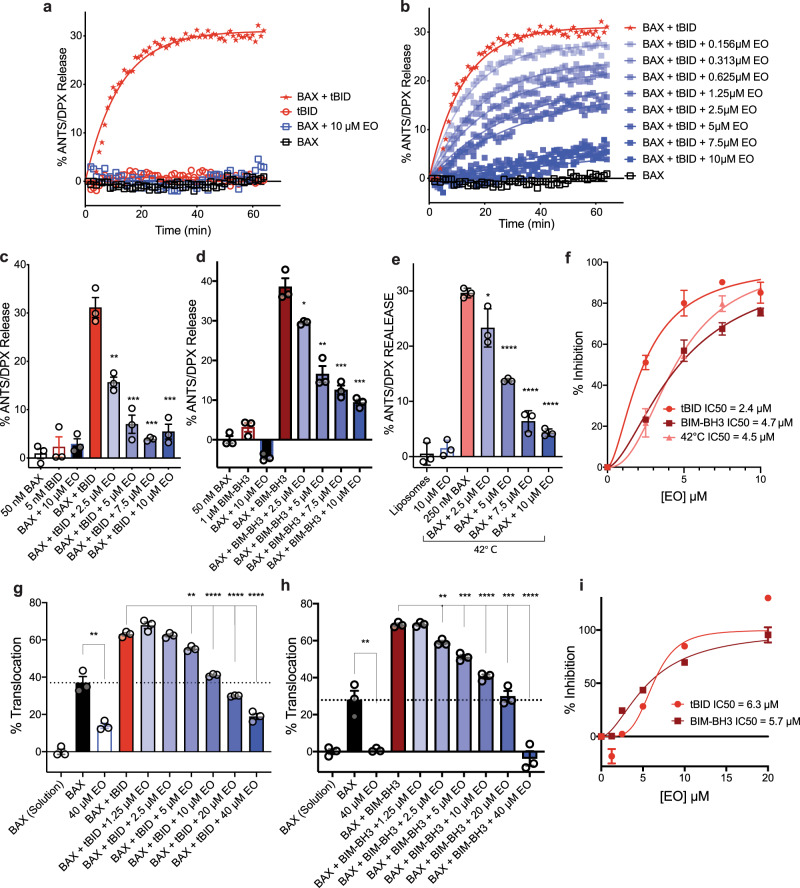
Eltrombopag inhibits BAX activation. **a**–**e** BAX-mediated membrane permeabilization assay using liposomes with 50 nM BAX and 5 nM tBID (**a**–**c**), 50 nM BAX and 1 µM BIM–BH3 (**d**), or 250 nM BAX at 42 °C (**e**), each at 30 min. Data are representative of three independent experiments, each *n* = 3 ± SEM. **f** Summary percentage inhibition curve for all liposomal release stimuli with IC_50_ included for clarity. Data are representative of three independent experiments, each *n* = 3 ± SEM. **g**, **h** Membrane translocation assay using NBD-labeled BAX (800 nM) activated by tBID (200 nM) (**g**) and BIM–BH3 (1 μM) (**h**) each at 120 min. Data are representative of three independent experiments, each *n* = 3 ± SEM. **i** Summary percentage inhibition curve for all BAX translocation stimuli with IC_50_ included for clarity. Data are representative of three independent experiments, each *n* = 3 ± SEM. Two-sided *t* test, ****P < 0.0001; ***P < 0.001; **P < 0.01; *P < 0.05; ns, *P* > 0.05. (left to right *P* values were 0.0027, 0.0010, 0.0002, 0.0006 for (**c**), 0.0154, 0.0019, 0.0005, 0.0002 for (**d**), 0.0379, 0.000008, 0.00004, 0.000002 for (**e**), 0.0029, 0.0025, 0.00002, 0.000003, 0.00002 for (**g**), 0.0056, 0.0014, 0.0002, 0.00004, 0.0002, 0.00006 for (**h**)). Source data for this figure is provided.

We evaluated EO’s capacity to modulate BAX activity using liposomal release assays. EO exhibited no ability to activate recombinant BAX in concentrations even up to 10 μM (Fig. 2a). Instead, EO was able to inhibit both tBID- and BIM–BH3-mediated BAX activation (Fig. 2b–d and Supplementary Fig. 2a–c). Based on the competitive nature of EO binding to the N-terminal BAX trigger site, we predicted that EO inhibition of BAX activity would be dependent on the BH3-only activator concentration. Indeed, inhibition of tBID-mediated BAX activation by EO was inversely proportional to the concentration of the tBID activator, consistent with competitive inhibition of BH3-activator binding (Supplementary Fig. 2d–f). Furthermore, EO was capable of inhibiting heat-induced BAX activation, suggesting that EO can stabilize inactive BAX in addition to blocking the BAX activation site from BH3-mediated activators (Fig. 2e and Supplementary Fig. 3a–c). EO exhibited similar low micromolar potency in inhibiting BAX activity in liposomal release assay against all stimuli (tBID IC_50_ = 2.4 µM, BIM–BH3 IC_50_ = 4.7 µM, heat IC_50_ = 4.5 µM). (Fig. 2f and Supplementary Fig. 3c).

Prior to permeabilizing membranes, activated BAX must first translocate to the membrane^37^. To explore this earlier step in BAX activation, we utilized an NBD-fluorescence based translocation assay (Fig. 2g–i and Supplementary Fig. 3d, e). EO was capable of inhibiting tBID- and BIM–BH3-induced BAX translocation with comparable potency to that of inhibiting liposomal release (tBID IC_50_ = 6.3 µM, BIM–BH3 IC_50_ = 5.7 µM). Similarly, EO was capable of inhibiting heat-induced auto-translocation of BAX, suggesting that EO binding stabilizes a soluble conformation of BAX (Supplementary Fig. 3f, g). One of the earliest conformational changes of BH3-mediated BAX activation is the opening of the α1–α2 loop and the exposure of an N-terminal 6A7 epitope^16,18^. In an immunoprecipitation assay using the anti-6A7 epitope-specific antibody, BIM–BH3-induced 6A7 exposure as previously shown, but it was inhibited by the presence of EO (Supplementary Fig. 3h). Taken together, the data suggest EO inhibits BAX activation process at an early stage by blocking activator binding and stabilizing a soluble inactive form of BAX.

### Eltrombopag forms unique contacts at the BAX trigger site

**Fig. 3:**
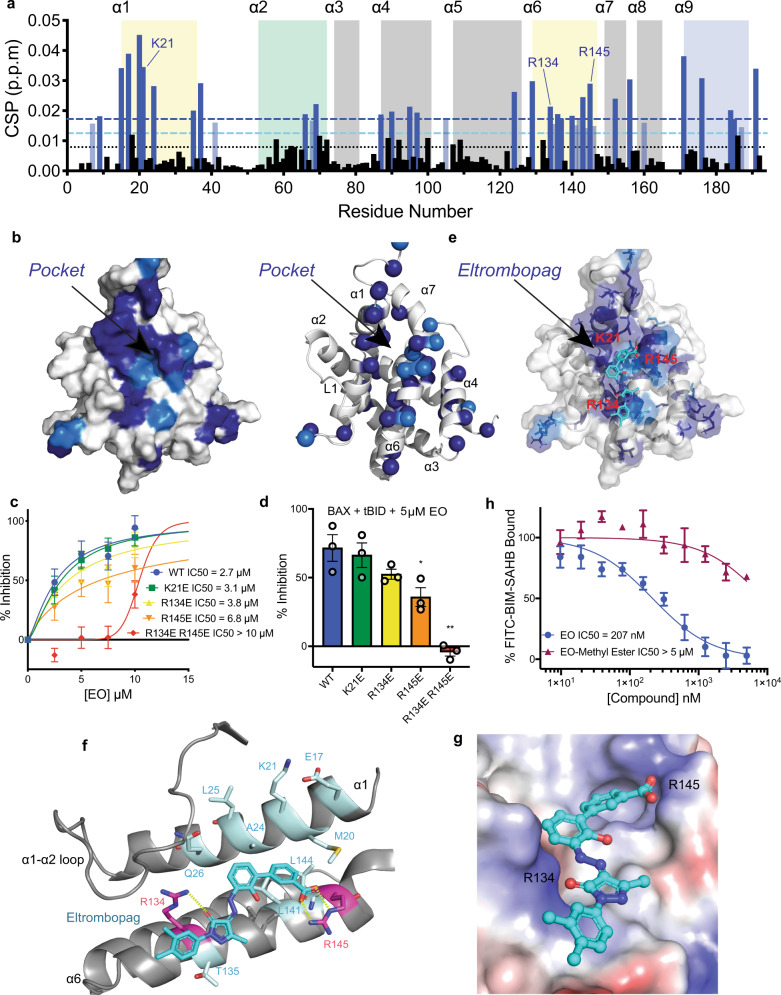
Eltrombopag binds the BAX trigger using unique contacts. **a** Measured chemical shift perturbations (CSPs) of ^15^N-labeled BAX in the presence of 1:2 BAX:EO are plotted as a function of BAX residue number. Residues with chemical shift perturbations over the significance threshold or two times the significance threshold are labeled light blue or dark blue, respectively. The black dotted line represents the average CSP. Residues associated with the N-terminal trigger site, BH3 domain, canonical site, and transmembrane domain are highlighted in yellow, green, gray, and blue, respectively. Basic residues of the N-terminal trigger site are labeled for clarity. Data are representative of three independent experiments. **b** Mapping of residues undergoing significant CSPs to the surface and the ribbon structure of BAX (PDB: 1F16: 10.2210/pdb1F16/pdb). Residues with significant CSPs cluster on the N-terminal trigger site of BAX surrounding a hydrophobic pocket formed by α1 and α6. **c**, **d** Percent inhibition of BAX-mediated membrane permeabilization assay using liposomes with 250 nM BAX and 5 nM tBID with various trigger site mutants, dose-response IC_50_ (**c**) and bar graph for 5 µM EO (**d**) are shown for clarity. Data are representative of two independent experiments, each *n* = 3 ± SEM. **e** Transparent surface with ribbon representation of the EO binding site as determined by NMR data and docking. **f** Close up view of the EO-binding site with residues determined by NMR data forming hydrophobic contacts with EO are highlighted in cyan and residues forming specific interactions, R134 and R145, are highlighted in red. **g** BAX electrostatic surface representation highlighting positive (blue) and negative (red) charge as a gradient and hydrophobic surfaces as gray. **h** Competitive fluorescence polarization binding assay of EO and inactive EO-methyl ester analog. Data are representative of three independent experiments, *n* = 3 ± SEM. Two-sided *t* test, ****P < 0.0001; ***P < 0.001; **P < 0.01; *P < 0.05; ns, *P* > 0.05. (left to right *P* values were 0.0395, 0.0017 for (**d**)). Source data for this figure is provided.

While a high-resolution structure of the BAX-EO complex would be ideal, such structures have been elusive due to technical challenges resulting from the dynamic BAX structure. To determine the binding site of EO, we performed 2D ^1^H–^15^N heteronuclear single quantum coherence (HSQC) NMR analysis with ^15^N-labeled BAX. EO titration shifted select cross-peaks of corresponding BAX residues in the NMR spectra. Analysis of the chemical shift perturbations (CSPs) of BAX in the presence of EO indicated small and specific shifts, as with previous NMR studies of BAX^17,18,33,34,38,39^. Significant CSPs were localized predominantly to the N-terminal BAX trigger site, specifically the N-terminal region of α1 and the length of α6 (Fig. 3a). Mapping of the CSPs onto the inactive BAX structure revealed that CSPs localizing to the BAX trigger site coalesce to form a contiguous surface with a shallow hydrophobic pocket between α1 and α6 (Fig. 3b). Additional CSPs corresponding to residues in adjacent helices to the trigger site, α4 and α7 but also distant at the C-terminal α9 were observed (Fig. 3a, b). Previous crystal structures of the inactive BAX mutants P168G and W139A suggested that binding at the N-terminal trigger site may modulate BAX activity via local conformational changes at α4, α7, and α9^40^. NMR analysis of BAX activation with BIM–SAHB and small-molecule trigger site activators also highlighted allosteric sensing in α4, α7, and α9^17,33^. The observed distal CSPs could correspond to similar conformational effects from the binding of EO to the BAX trigger site. Notably, we observed few chemical shift perturbations in N-terminal α1–α2 loop residues, suggesting that its structure remains largely unchanged upon EO binding (Fig. 3a, b). This is in direct contrast with BIM–SAHB and BTSA1 binding, which induce significant CSPs in α1–α2 loop residues and corresponding with the displacement of the α1–α2 loop from the trigger site, a critical step in the activation of BAX^17,33,34^.

We next performed CSP-guided molecular docking of EO to the BAX surface to determine the binding pose of EO to the inactive BAX structure (PDB: 1F16: 10.2210/pdb1F16/pd)^16^. We predicted that the negatively charged EO carboxylate would form a favorable interaction with one of the three basic residues at the EO-binding interface highlighted by the NMR data, K21, R134, or R145, all of which exhibited significant CSPs (Fig. 3a). We, therefore, performed molecular docking of EO to a site centered about residues K21, R134, and R145. We performed induced-fit docking (IFD) with Schrödinger software using a largely extended surface of BAX at the trigger site to account for potential ambiguity with the NMR data and exhaustively consider possible binding modes and the associated local conformational changes of α1, α6, and α1–α2 loop residues. The IFD yielded several poses which featured ionic interactions between the EO carboxylate and K21, R134, or R145 as expected (Supplementary Fig. 4a, b).

We set out to determine which EO pose was appropriate by comparing BAX trigger site mutants that would eliminate one of the three basic trigger site residues, K21E, R134E, or R145E, to wild-type (WT). All tested mutants exhibited identical retention time in size-exclusion chromatography analysis, suggesting no effect on BAX folding and had comparable purity and molecular weight to WT BAX as determined by SDS-PAGE (Supplementary Fig. 4c, d). In liposomal release assays, all of the BAX mutants were functional, although R134E and R145E exhibited lower ANTS/DPX release in response to tBID activation (Supplementary Fig. 4e). Of the mutants tested, only BAX R145E exhibited a reduced inhibition in response to EO, with an IC_50_ more than double that of BAX WT (Fig. 3c, d). Consistently, HSQC-NMR analysis of ^15^N-labeled BAX R145E in the presence of EO exhibited few non-localized CSPs, indicating a significant reduction in EO-BAX affinity (Supplementary Fig. 4f, g). Notably, BAX K21E exhibits reduced activation in response to BIM–BH3, BAM7, and BTSA1 activators but not reduced inhibition in response to EO^17,18,33,34^. This highlights how unique contacts at the trigger site could determine whether compounds will behave as BAX activators or inhibitors.

The loss of EO-mediated BAX inhibition with the R145E mutant suggests that EO forms a critical interaction via the anionic carboxylate with BAX R145. With this known, we reevaluated the EO docking poses and analyzed the top pose featuring an ionic interaction between the EO carboxylate and the side chain of R145 (Fig. 3e–g). In addition to the ionic interaction, this pose also features hydrophobic interactions between the biphenyl moiety of EO and the hydrophobic pocket formed by residues L24, M137, G138, and L141 between α1 and α6. Furthermore, the docking pose features contacts at the N-terminal of α6 unique to poses possessing an ionic interaction with R145 (Supplementary Fig. 4a, b). Computational prediction of BAX-binding energy for EO, BAM7, and BTSA1 accurately predicted this pose of EO to be the highest affinity and correlated to the observed IC50 values in FPA (Supplementary Fig. 4h). Of particular note is a hydrogen bond between the R134 side chain and the carbonyl of the pyrazolone core of EO, which could potentially explain the slight trend toward weaker inhibition of BAX R134E (Fig. 3c, d). HSQC-NMR analysis of ^15^N-labeled BAX R134E in the presence of EO exhibited clustering of CSPs to the trigger site, albeit with reduced CSPs compared to WT BAX (Supplementary Fig. 4i–j). We, therefore, hypothesized that the double-mutant BAX R134E R145E would markedly reduce EO inhibition of BAX. Indeed, EO exhibited clearly weak inhibition of BAX R134E R145E with an IC_50_ > 10 µM (Fig. 3c, d). Consistently, in the presence of EO, this double mutant also exhibited minimal CSPs of ^15^N-labeled BAX by HSQC-NMR studies and markedly reduced affinity as determined by MST (Supplementary Fig. 5a–c and Fig. 1a, c).

To further probe the specificity of EO for the BAX trigger site and the critical R145 ionic interaction, we utilized an EO analog featuring a methyl ester (EO-Methyl Ester; **4**) in place of the carboxylic acid (Supplementary Fig. 5d). The addition of a methyl group eliminates the anionic carboxylate as well as adds steric bulk at the site of the critical R145 interaction (Fig. 3e–g). EO-Methyl Ester exhibited minimal competition of FITC-BIM–SAHB by FPA (IC_50_ > 5 µM), indicating a dramatically diminished binding affinity (Fig. 3h). Consistently, EO-Methyl Ester induced minimal CSPs of ^15^N-labeled BAX by HSQC-NMR studies and exhibited significantly diminished inhibition of BAX in liposomal release assays (Supplementary Fig. 5e–h). Taken together, the data show that EO binds to the N-terminal trigger site of BAX forming contacts with a shallow hydrophobic pocket between α1 and α6, interacting predominantly with α6 residues. Notably, this pocket is formed adjacent to the α1–α2 loop, which interacts with residues of α1 and α6 and it is not disturbed by EO binding. Docking, mutagenesis, and the EO-Methyl Ester suggest EO makes a critical contact with R145 via the anionic carboxylate as well as a secondary hydrogen bond with R134 via the pyrazolone carbonyl.

### Stabilization of inactive BAX by Eltrombopag

**Fig. 4:**
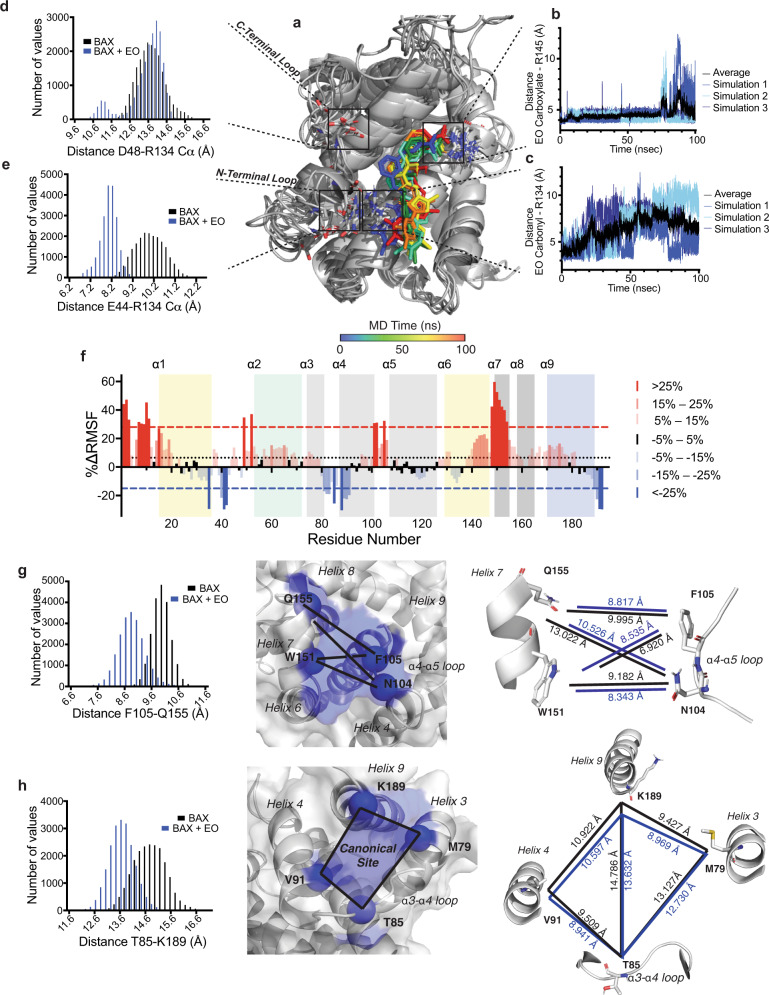
Eltrombopag stabilizes inactive BAX structure. **a** Overlay of structures of BAX-EO complex from 10 nsec intervals from 0 to 100 nsec molecular dynamics (MD) simulation. EO color spectrum corresponds to time as described, BAX ribbon structure is colored gray with residues of interest represented as sticks for clarity. **b**, **c** Distance relative to the time of EO carboxylate-R145 (carbonyl carbon-ζ-carbon) (**b**) and EO pyrazolone carbonyl-R134 (carbonyl oxygen- ζ-carbon) (**c**). Blue shades represent individual MD simulation distances, and black represents mean of *n* = 3 simulations. **d**, **e** Histogram representation of α-carbon distance frequency during MD simulation between R134 and negatively charged residues D48 (**d**) and E44 (**e**) on α1–α2 loop. Data represent a mean of *n* = 3 for both BAX and BAX-EO. Data represent a mean of *n* = 3 simulations for both BAX and BAX-EO. **f** Percentage change in root-mean square fluctuation (RMSF) of BAX-EO versus BAX is plotted with respect to BAX residue number. The color gradient is representative of change in RMSF, with blue and red corresponding to decrease and increase in RMSF, respectively. Data represent a mean of *n* = 3 simulations for both BAX and BAX-EO. The average percentage change is represented by the dotted black line, with ±SD represented as red and blue dashed lines. Residues associated with the N-terminal trigger site, BH3 domain, canonical site, and transmembrane domain are highlighted in yellow, green, gray, and blue, respectively. **g** Changes in structure and dynamics of α7/α4-α5 loop interface: representative α-carbon distance frequency histogram for F105-Q155 (left), transparent surface with ribbon representation of α7/α4-α5 loop interface with residues of note highlighted in blue (center), and graphical representation of distances between residues at α7/α4-α5 loop interface (right). **h** Changes in structure and dynamics of the canonical site opening formed by α3, loop 3, α4, and α9: representative α-carbon distance frequency histogram for T85-K189 (left), transparent surface with ribbon representation of canonical site opening with residues of note highlighted in blue (center), and graphical representation of distances between residues at the canonical site opening (right). Distances represent the mean difference of *n* = 3 BAX and BAX-EO MD simulations (**g**, **h**), Distance plotted with respect to time and distance frequency histograms for all distances are available in supplementary figures as referenced. Source data for this figure is provided.

Our data suggest that the N-terminal BAX trigger site, which has been established as the binding site for both BH3-only proteins and small-molecule activators, could also be an inhibitory site. NMR and mutagenesis data indicated that the distinct contacts and binding mode of EO may be responsible for the inhibitory activity of EO. To explore how EO can accomplish inhibition of BAX conformation and activity, we performed three independent molecular dynamics (MD) simulations of the BAX-EO complex and of the inactive BAX structure. The overall structure of BAX was maintained in all six simulations as determined by similar RMSD and radius of gyration in the presence and absence of EO (Supplementary Fig. 6a–h). In the three MD simulations of the BAX-EO complex, EO remains in a stable conformation (Fig. 4a and Supplementary Fig. 6a–f). The distance between R145 and the EO carboxylate remains stable throughout the simulation (Fig. 4b). The interaction between R134 and the EO carbonyl is noticeably more dynamic, however the two groups remain in close proximity throughout the simulation (Fig. 4c). The EO-BAX distances strongly support the mutagenesis data and binding mode wherein EO forms a critical interaction at R145 and a secondary weaker interaction at R134.

HSQC CSPs suggested that EO does not cause significant conformational changes to α1–α2 loop, in contrast with other trigger site binders, BTSA1 and BH3 peptides^33,34,41^. In the unbound BAX structure, R134 on α6 sits in close proximity to E44 and D48 on α1–α2 loop^16^. The distance between R134 and D48 is approximately equal for both the BAX and BAX-EO simulations (Fig. 4d and Supplementary Fig. 7a, b). However, R134 and E44 remain in closer proximity in simulations of the BAX-EO complex than in BAX alone (Fig. 4e and Supplementary Fig. 7c, d). Furthermore, the BAX-EO simulations displayed a narrower distribution of distances indicating reduced conformational flexibility in the N-terminal region of α1–α2 loop. Notably, the few CSPs observed on α1–α2 loop were towards the N-terminal region (Fig. 3a).

To further explore potential conformational changes associated with EO binding to BAX, we analyzed the percentage change in root-mean-square fluctuation (RMSF), a measure of the dynamics of each residue in the BAX structure (Fig. 4f and Supplementary Fig. 8). Although no net change in RMSD was observed, discrete clusters of residues exhibited increases and decreases in dynamics. Residues towards the N and C-terminus of α1–α2 loop exhibit reduced and increased RMSF, respectively, as expected based on α1–α2 loop distances to R134. Two additional regions exhibited dramatic changes in RMSF. The α4–α5 loop and helix α7 exhibit an increase in RMSF whereas α3–α4 loop and the C-terminal helix α9 exhibit a decrease in RMSF.

The interface between α4–α5 loop and α7 has been identified as an important site for communication between the N-terminal trigger site and the C-terminal canonical site^18,40^. Crystal structures of inactive BAX mutants (P168G and W139A) display changes in the interface of α4–α5 loop and α7, with notable changes in the conformation of F105 and W151. We measured the distances between α4–α5 loop and α7 and observed reduced distances between most of the residues in the presence of EO with the exception of the distance between F105 to W151, which increases (Fig. 4g and Supplementary Fig. 9). Furthermore, we observed changes in the distances between R89 and W139 on α4 and α6, respectively, as well as in the distances between R89 and F93 on α6, both of which showed changes in the inactive BAX crystal structures^40^ (Supplementary Fig. 10). The MD data strongly agree with the crystallographic structures of inactive BAX mutants and point to a potential mechanism by which binding at the trigger site propagates conformational changes throughout BAX.

The α3–α4 loop and α9 form what can be considered as the opening of the canonical site. In order for BAX to translocate to the mitochondria, α9 must partially dissociate from the canonical site of BAX^18,19,40^. To evaluate this, we measured the distances between four residues forming the boundaries of the opening to the canonical site (Fig. 4h and Supplementary Fig. 11). All of the distances measured were reduced. By approximating the canonical site opening as two triangles, we were able to calculate the approximate canonical site opening area of BAX as 113 Å^2^ and the BAX-EO complex as 102 Å^2^, a reduction of ~9%. Taken together, the MD data suggest that EO binding at the BAX trigger site induces direct and distal conformational changes consistent with stabilization of the inactive soluble BAX structure. These include changes in the interfaces of α4 and α6 as well as α4–α5 loop and α7, which may allosterically couple the trigger site to α9 and the canonical site.

**Fig. 5:**
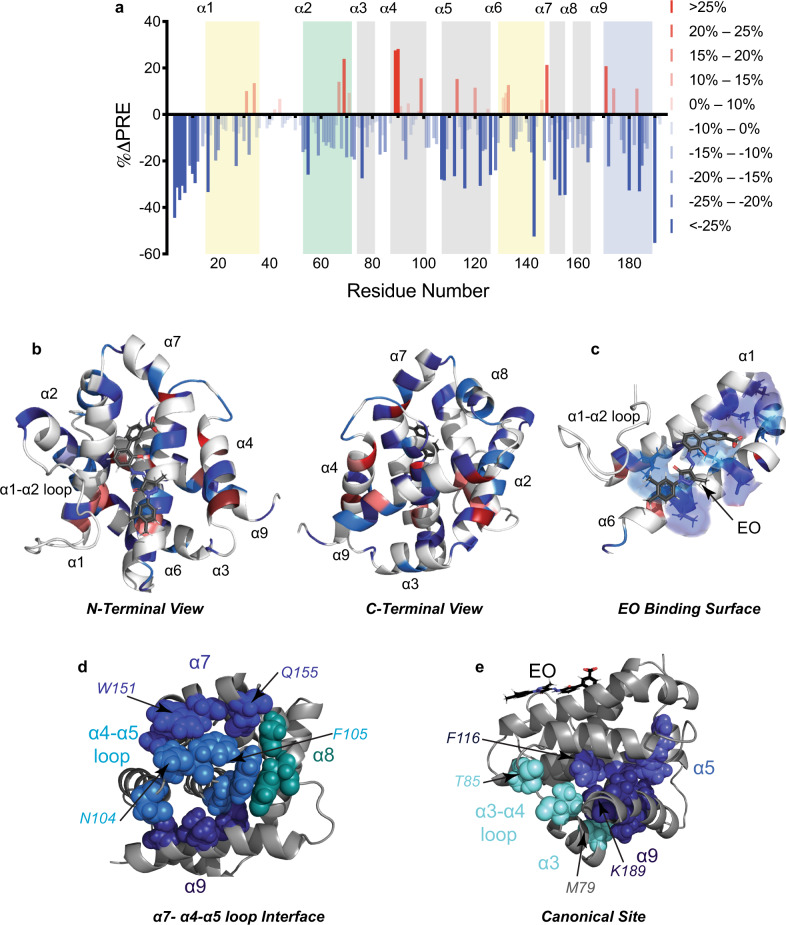
NMR-based evidence of Eltrombopag-mediated BAX inhibition. **a** EO-induced differences in peak intensity ratio (PRE) of peaks in the presence and absence of the soluble paramagnetic probe hydroxyl-TEMPO. Percentage change in PRE is plotted with respect to BAX residue number. Residues that exhibit increased or decreased PRE are labeled in shades of red and blue, respectively, the color gradient corresponding to the figure key. Residues associated with the N-terminal trigger site, BH3 domain, canonical site, and transmembrane domain are highlighted in yellow, green, gray, and blue, respectively. **b** Ribbon representation of BAX-EO complex with mapping of residues undergoing significant differences in PRE as shown in (**a**). **c** Ribbon representation of EO-binding site with residues undergoing significant change in PRE colored corresponding to (**a**) and represented as a transparent surface with stick representation of residues. **d**, **e** Cluster of residues about the α7/α4–α5 loop interface (**d**) and canonical site (**e**) which exhibited reduced PRE in the presence of EO. Colors correspond to the location of the residue within the tertiary structure of BAX as indicated by the figure labels. Source data for this figure is provided.

To independently assess the results of MD simulations, we measured paramagnetic relaxation enhancement (PRE) effects on ^15^N-labeled BAX caused by a soluble paramagnetic probe, hy-TEMPO, in the presence and absence of EO. The hy-TEMPO probe is a small sparsely functionalized molecule that can bind nonspecifically to solvent-exposed surfaces and pockets on the surface of BAX (Supplementary Fig. 12). We observed that the presence of EO altered the PRE effects not only by directly blocking hy-TEMPO binding to the trigger site but by altering the surface topology of BAX (Fig. 5a). Mapping these changes to the surface of BAX revealed that EO binding protected the trigger site residues in direct contact with EO based on the docking pose, particularly around the hydrophobic pocket formed between α1 and α6 (Fig. 5b, c). As expected, PRE effects on α1–α2 loop were unchanged in the presence of EO, consistent with this loop remaining closely associated with the trigger site. Furthermore, reduction in PRE effects were observed on residues surrounding the interface of α7 and α4–α5 loop as well as internal residues of the canonical sites, such as α3, α5, and α9 (Fig. 5d, e). This reduction in PRE effects at the interface of α7 and α4–α5 loop is consistent with the closer association predicted by MD simulations (Fig. 4). Furthermore, the reduction in PRE effects on internal canonical site residues is consistent with stabilization of α9 binding at the canonical site and a narrowing of the canonical site opening as suggested by MD simulations (Fig. 4). The PRE effects on these regions strongly corroborate our findings from biochemical, NMR, and molecular dynamics data and further support that binding events at the BAX trigger site can induce distal conformational changes in the canonical site via changes in α4, α4–α5 loop, and α7, which result in inhibition of BAX activation and stabilization of the inactive BAX structure.

### Eltrombopag inhibits BAX-mediated apoptosis

**Fig. 6:**
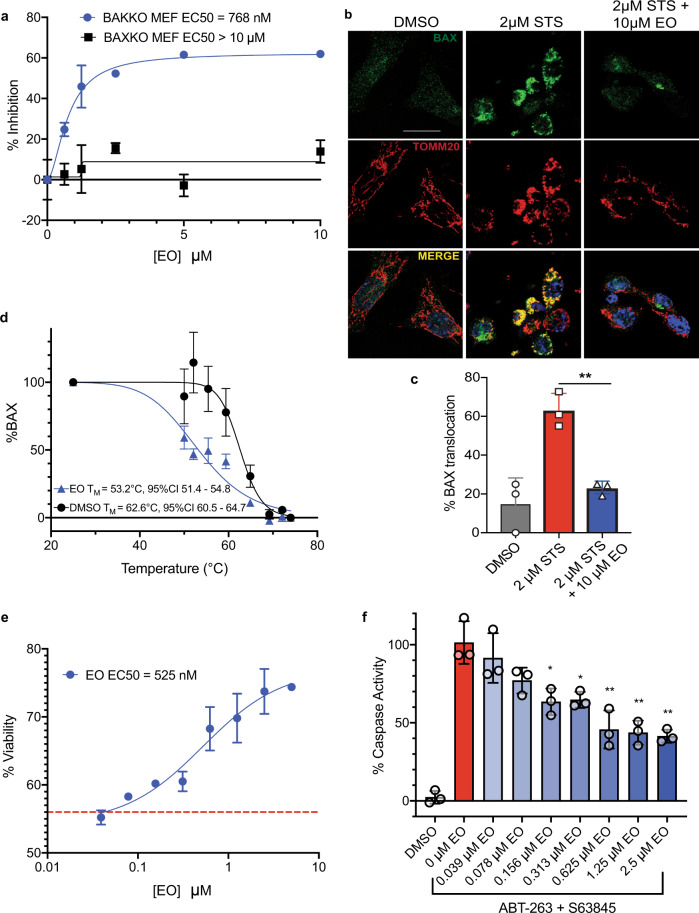
Eltrombopag inhibits BAX-mediated cell death. **a** Dose-response EC50 curve for percentage inhibition of BIM–BH3-induced cytochrome *c* release by EO in BAK KO (blue) and BAX KO (black) mouse embryonic fibroblasts with EC50 shown for clarity. Data represent mean of *n* = 3 ± SEM and are representative of three independent experiments. **b** Confocal micrographs of BAK KO MEFs treated with DMSO, 2 μM STS for 4.5 h without or with 10 μM EO 6.5 h, respectively. BAX translocation is based on antibody-based detection of BAX and mitochondrial protein TOMM20. Representative confocal micrographs from three independent biological experiments. Scale bar, 20 μm. **c** Quantification of BAX translocation (% of cells with BAX foci colocalizing with TOMM20 foci) in BAK KO MEFs induced by 2 µM staurosporine (STS) and inhibited by 10 µM EO. Data represent ±SEM of three independent biological replicates. Two-sided *t* test, ****P < 0.0001; ***P < 0.001; **P < 0.01; *P < 0.05; ns, *P* > 0.05. **d** Cellular thermal shift assay (CETSA) BAX melting curves for BAK KO MEFs treated with vehicle or 10 µM EO. Data represent mean of *n* = 4 ± SEM independent experiments. **e** Viability assay of 3T3 cells upon treatment with 1 µM ABT-263 and 1 µM S63845 in the presence or absence of various doses of EO for 24 h. Viability upon ABT-263 and S63845 combination in the absence of EO is indicated by the red dashed line. Data represent mean of *n* = 3 ± SEM and are representative of three independent experiments. **f** Caspase-3/7 assay of 3T3 cells upon treatment with 1 µM ABT-263 and 1 µM S63845 in the presence or absence of various doses of EO for 4 h . Data represent mean of *n* = 3 ± SEM and are representative of three independent experiments. Two-sided *t* test, ****P < 0.0001; ***P < 0.001; **P < 0.01; *P < 0.05; ns, *P* > 0.05. (left to right *P* values were 0.0019 for (**c**), 0.0151, 0.0122, 0.0062, 0.0031, 0.0019 for (**f**)). Source data for this figure is provided.

It is important to note that thrombopoietin (THPO)-receptor agonist activity of EO is highly specific to the human and chimpanzee THPO-receptors, making mouse cell lines ideal for studying EO modulation of BAX-dependent activity independent of THPO-mediated effects^42^ (Supplementary Fig. 13a). First, we evaluated mitochondrial cytochrome *c* release, a hallmark of BAX activation and BAX-dependent apoptosis. BIM–BH3-induced release of cytochrome *c* was significantly inhibited by EO in BAK KO (BAK^−/−^) MEFs providing direct evidence that EO can inhibit BAX-dependent cytochrome *c* release (Fig. 6a). EO had no such effect in BAX KO (BAX^−/−^) MEFs, strongly supporting BAX specificity (Supplementary Fig. 13b, c). BAX KO and BAK KO MEFs exhibited similar sensitivity to BIM–BH3-induced cytochrome *c* release (Supplementary Fig. 13d). Next, we evaluated mitochondrial translocation of cytosolic BAX upon treatment with either BIM–BH3 or staurosporine (STS) in BAK KO MEFs, and we determined that EO is capable of inhibiting BAX translocation (Fig. 6b, c and Supplementary Fig. 13e), consistent with in vitro results (Fig. 2g–i and Supplementary Fig. 3d–g). Accordingly, EO inhibited STS-induced apoptosis mediated by caspase-3/7 activity in MEFs expressing only BAX, but it had no effect is MEFs expressing only BAK (Supplementary Fig. 13f, g). To confirm whether these effects are linked to direct target engagement of BAX, we performed Cellular Thermal Shift Assay (*CETSA*) in BAK KO MEFs. CETSA showed that EO indeed binds BAX in cells by lowering its T_M_ by 9 °C (Fig. 6d and Supplementary Fig. 14a). Interestingly, previous studies also have demonstrated that inactive BAX mutants can display dramatically reduced T_M_ despite their resistance to activation by BH3 activators, highlighting a critical distinction between the controlled conformational changes of BAX activation and protein unfolding^41,43^. Furthermore, recombinant BAX displayed a comparable reduction in T_M_ in the presence of EO, strongly supporting the CETSA observations (Supplementary Fig. 14b, c). Finally, we determined if EO is capable of rescuing cell death in cells expressing both BAX and BAK. We treated 3T3 cells, a murine fibroblast cell line, with a combination of clinical BH3-mimetics ABT-263 (Navitoclax) and S63845, which only in combination cause significant cytotoxicity in fibroblast cells (Supplementary Fig. 14d). Strikingly, cells treated with EO exhibited a dose-dependent rescue of cell viability as well as a corresponding significant reduction of apoptosis mediated by caspase-3/7 activity (Fig. 6e, f). Although EO does not inhibit BAK-mediated cytochrome *c* release and apoptosis, it is capable of inhibiting cell death in cells expressing both BAK and BAX. It could be that in these cells, BAX is predominant in mediating apoptosis or EO inhibition of BAX frees more anti-apoptotic BCL-2 proteins to inhibit BAK in response to BH3 mimetics^30,31^. While we cannot definitely rule out other potential intracellular targets, our data strongly suggest EO targeting BAX in cells to inhibit cell death.

## Discussion

**Fig. 7:**
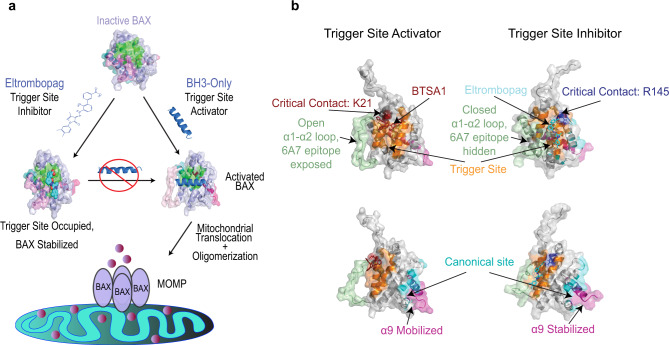
Eltrombopag inhibits BAX activation by a unique twofold mechanism. **a** Eltrombopag’s binding to the BAX trigger site competes off BH3-only activator proteins of BAX and stabilizes an inactive structure of BAX resulting in inhibition of BAX activation, mitochondrial translocation, and BAX-mediated MOMP. **b** BAX surface representations demonstrating distinct binding modes and associated conformational effects by the BAX trigger site inhibitor, Eltrombopag, and the BAX trigger site activator, BTSA1.

Understanding the mechanism of BAX activation enables a deeper understanding of the critical function of BAX in apoptosis signaling and related pathways (e.g., mitochondrial-driven necrosis, mitochondrial dynamics) as well illuminates previously unknown targets and means to modulate BAX activity with drugs^28,29,31,34^. Here, our studies have identified EO, as a potent binder to the BAX trigger site and an effective BAX inhibitor (Fig. 7a). EO inhibits BAX activation by a unique two-fold mechanism. BAX inhibition by EO is dependent on the concentration of EO, BAX, and BH3 activators, and EO directly engages the BAX trigger site, consistent with a direct competitive mechanism. Unlike trigger site activator small molecules and peptides that bind and displace loop α1-α2 from the trigger site as the critical first step in BAX activation, EO binds the BAX trigger site in a unique conformation that maintains the α1-α2 loop in an inactive closed conformation^18,30,33,34^. Likewise, BAX mutations at the trigger site that do not affect loop α1-α2 conformation can also inhibit BAX activation^40^. Furthermore, EO inhibits BAX by inducing discrete conformational changes in previously reported regions of allosteric communication such as α7/α4-α5 loop which stabilizes α9 in the canonical site^40–43^. Thus, our data suggest a unique mechanism of BAX inhibition by EO that directly competes BH3-only proteins binding to BAX maintaining α1–α2 loop in an inactive closed conformation and simultaneously promoting allosteric conformational changes that stabilize the inactive soluble BAX structure.

The N-terminal BAX trigger site has been established as an important binding site for BH3-only proteins and small-molecule BAX activators that induce conformational changes throughout the BAX structure necessary for mitochondrial translocation, dimerization/oligomerization, and mitochondrial outer membrane permeabilization^10,17,19,33,34,40,41,43,44^. This study provides further evidence that BH3-only proteins such as BID and BIM use the trigger site surface to induce BAX conformational activation. We identified EO by substructure similarity search of known small-molecule BAX activators. Despite some similar structural features to BAX activators, EO engages the trigger site with a unique binding mode distinct from BAX activators, using hydrophobic interactions with a shallow hydrophobic groove formed by residues of α6, α1, and the closed α1–α2 loop, and hydrogen bonds with R145 and R134 of α6. Here, we show that the BAX trigger site can also serve as a site of inhibition by small molecules and it can interfere with the early stages of BAX activation, before the disengagement of the α9 from the canonical groove and BAX mitochondrial translocation (Fig. 7b). Accordingly, our study offers a blueprint for rational drug design of a distinct class of BAX inhibitors using EO’s mechanism of action of targeting BAX.

Our data contribute to the growing body of work highlighting bifunctional allosteric communication between the BAX trigger site and canonical site. Allostery has been proposed with activator BH3 peptides and small molecules such as BAM7 and BTSA1 that engage the BAX trigger site and promote conformational changes leading to the release of α9 from the opposite surface of BAX^18,33,34^. Allosteric BAX inhibitors (BAIs) and fragments that sensitize BAX to activation bind to adjacent sites while exhibiting opposite functional effects^30,45^. Furthermore, the BAX sensitizing fragments and the cytomegalovirus vMIA peptide compete for an identical binding site, yet the vMIA peptide inhibits BAX activity^38,45^. Synthetic antibodies recognizing the N-terminal BAX trigger site in a way that prevents opening of loop α1-α2 have also served to inhibit BAX activation by blocking activator binding^44^. Furthermore, the BCL-2 BH4 domain inhibits BAX by binding to a unique site on the surface directly between the N-terminal trigger site and vMIA site^39^. Lastly, antibody 3C10 has an inhibitory effect to BAX by engaging the α1–α2 loop and favoring the allosteric sequestration of α9 in the canonical groove^40,46^. Therefore, it is possible that most if not all of the BAX surface could serve as a site of activation or inhibition given the appropriate interactions with a small molecule, peptide, or antibody/protein. Fine-tuning of the pro-apoptotic function of BAX via multiple structural mechanisms is critical in a physiologic context but could potentially be exploited pharmacologically to induce or inhibit cell death.

To our knowledge, EO is the first FDA-approved molecule with the ability to modulate BAX activity directly. The fact that EO is a well-tolerated orally bioavailable molecule inspires confidence in the potential of BAX inhibition as a therapeutic strategy for diseases of aberrant cell death. Interestingly, platelets isolated from patients treated with EO exhibited increased resistance to ABT-263-induced cell death^47^. This was not observed in patients treated with the fusion protein thrombopoietin receptor agonist romiplostin^47^, suggesting it is possible that EO inhibition of BAX contributes to its therapeutic activity. Although increased platelet levels after several days of continuous treatment with EO could prevent the repurposing of EO as a BAX inhibitor, evaluation of EO’s ability to inhibit clinically relevant BAX-mediated cell death induced by acute apoptotic stimuli should be the subject of a future investigation. Furthermore, EO may serve as a powerful chemical tool for investigating the role of BAX in a variety of homeostatic and pathologic conditions and for drug design tailored to BAX inhibition.

## Methods

### Reagents

Hydrocarbon-stapled peptides corresponding to the BH3 domain of BIM, BIM SAHB: FITC-Ahx-EIWIAQELRS5IGDS5FNAYYA-CONH, where S5 represents the non-natural amino acid inserted for olefin metathesis, were synthesized and purified at >95% purity by CPC Scientific Inc. Peptides corresponding to the BH3 domain of BIM, BIM–BH3, Ac-RPEIWIAQELRRIGDEFNAYYARR, was synthesized by GenScript at >95% purity. Recombinant tBID in >95% purity by SDS-PAGE under reducing conditions was purchased by R&D Systems (cat. # 882-B8-050). Eltrombopag (cat. # 100941) and Eltrombopag methyl ester (cat. # SC498745) were purchased from Medkoo Biosciences and Santa Cruz Biotechnology respectively and their molecular identity and purity >98% was confirmed by NMR. ABT-263 (cat. # S1001) in >99% purity, and S63845 (cat. # A8737) in 98% purity were purchased from Selleckchem, and APExBIO, respectively. Compounds were stored as powdered, reconstituted into 100% DMSO, and diluted as described.

### Production of recombinant BAX

Human full-length (1–192) wild-type BAX was cloned in pTYB1 vector (New England BioLabs) between the NdeI and SapI restriction sites. Mutations were generated using the QuickChange Lightning site-directed mutagenesis kit (Agilent). Recombinant proteins were expressed in BL21 (DE3) CodonPlus (DE3)-RIPL, grown in Luria Broth media and induced with 1 mM isopropyl β-d-1-thiogalactopyranoside. The bacterial pellet was resuspended in lysis buffer (20 mM Tris–HCl pH 7.6, 250 mM NaCl, 1 mM EDTA, and Roche complete EDTA-free protease inhibitor cocktail), lysed by high-pressure homogenization, and clarified by ultracentrifugation at 45,000×*g* for 45 min. The supernatant was applied to 5 ml of pre-equilibrated chitin beads (New England BioLabs) in a gravity-flow column, and washed with three column volumes of lysis buffer. BAX was cleaved by overnight incubation using 50 mM DTT in lysis buffer. Cleaved BAX was eluted with lysis buffer, concentrated with a Centricon spin concentrator (Millipore), and purified by gel filtration using a Superdex 75 10/300 GL, column (GE Healthcare Life Sciences), pre-equilibrated with gel filtration buffer (20 mM HEPES, 150 mM KCl, pH 7.2) at 4 °C. Fractions containing BAX monomer are pooled and concentrated using a 10-KDa cutoff Centricon spin concentrator (Millipore) for prompt use in biochemical and structural studies.

### Fluorescence polarization binding assays

Fluorescence polarization assays (FPA) were performed as previously described^34^. Direct binding isotherms of BIM–SAHB were measured by incubated FITC-BIM–SAHB (25 nM) with serial dilutions of full-length BAX alone or in the presence of 0.5 or 1 µM EO. Competition binding assays were performed by titrating EO into BAX (150 nM) and FITC-BIM–SAHB (25 nM). Measurements were taken at 10-min intervals over 60 min on a TECAN F200 PRO microplate reader. Reported curves represent a 10-min time point. K_D_ values and IC_50_ were determined using GraphPad Prism nonlinear fit four-parameter agonist or antagonist versus response with restraints for 100% and 0% bound calculated by the mP of saturated BAX + FITC-BIM–SAHB and FITC-BIM–SAHB alone.

### Microscale thermophoresis

Recombinant BAX C62S C126S S5C (4C), previously established for evaluating BAX^30^ binding compounds with MST, or BAX C62S C126S S5C R134E R145E (R134E R145E 4C) was labeled at cysteine using the Monolith Protein Labeling Kit Red Maleimide (NanoTemper Technologies) according to the instructions of the manufacturer^31^. Briefly, 10 µM protein was incubated with 0.9 equivalents of dye in MST buffer (100 mM potassium phosphate, pH 7.4, 150 mM NaCl) in the dark at room temperature (22–25 °C) for 1 h. Unreacted dye was quenched using 5 mM DTT and removed using the manufacturer provided buffer exchange column. To determine the K_D_ of BAX to EO, 50 nM labeled BAX was incubated with increasing concentrations of EO in MST buffer supplemented with 0.25% CHAPS. Samples were loaded into standard glass capillaries (Monolith NT.155 Capillaries) and analyzed by MST using a Monolith NT.115 Blue/Red, LED power, and IR laser power of 80%. The fraction bound and error was generated by NanoTemper software (MO.Affinity Analysis 3) and K_D_ values were determined using GraphPad Prism nonlinear fit four-parameter agonist versus response with restraints for 0 and 1 fraction bound.

### Liposomal permeabilization assay

Lipids (Avanti Polar Lipids) at the following ratio, phosphatidylcholine 48%, phosphatidylinositol 10%, dioleoyl phosphatidylserine 10%, phosphatidylethanolamine, 28%, and tetraoleoyl cardiolipin 4%, were mixed in a total of 1 mg, dried and resuspended in 10 mM HEPES, pH 7, 200 mM KCl, and 5 mM MgCl_2_ with 12.5 mM 8-aminonaphthalene-1,3,6-trisulfonic acid (ANTS) dye and 45 mM p-xylene-bis-pyridinium bromide (DPX) quencher (Molecular Probes) using a water bath sonicator. Liposomes were formed by extrusion of the suspension using Avanti Mini-Extruder (cat # 610000) with polycarbonate membranes of 0.1-µm pore size (Avanti Polar Lipids). ANTS/DPX encapsulated liposomes were purified from non-encapsulated ANTS/DPX by gel filtration of a 10 mL CL2B-Sepharose (GE Healthcare Life Sciences) gravity-flow column. BAX (50–250 nM) was combined with tBID, BIM–BH3, and EO at the indicated concentrations to a volume of 90 µL. Reactions were initiated by the addition of 10 µL of the encapsulated ANTS/DPX liposome stock. ANTS/DPX release was quantified based on the increase in fluorescence intensity that occurs when the ANTS fluorophore is separated from the DPX quencher upon release from the liposomes into solution. Fluorescence (*λ*ex = 355 nm and λem = 520 nm) was measured at 1 min of intervals at room temperature (22–25 °C) indicated using a Tecan Infinite M1000 plate reader. In the case of heat activation, reactions were set up as described in the absence of tBID or BIM–BH3, and experiments were recorded at 42 °C. The percentage release of ANTS/DPX at any given time point was calculated as percentage release = ((F − F_0_)/(F_100_ − F_0_))(100), where F_0_ and F_100_ are baseline and maximal fluorescence, respectively. Triton X-100 (1%) was used to determine the maximum amount of liposomal release per assay and is set to 100%.

### Liposomal translocation assay

Lipids (Avanti Polar Lipids) at the following ratio, phosphatidylcholine 48%, phosphatidylinositol 10%, dioleoyl phosphatidylserine 10%, phosphatidylethanolamine, 28%, and tetraoleoyl cardiolipin 4%, were mixed in a total of 1 mg, dried and resuspended in 10 mM HEPES, pH 7, 200 mM KCl, and 5 mM MgCl_2_. The resulting slurry was vortexed for 10 min and sonicated in a sonicating water bath for 10 min. Liposomes were formed by extrusion of the suspension using Avanti Mini-Extruder with polycarbonate membranes of 0.1-µm pore size (Avanti Polar Lipids) followed by passage through a CL2B-Sepharose column (GE Healthcare). Recombinant wild-type BAX was labeled at cysteine by overnight incubation at 4 °C with ten equivalents of iodoamino-NBD (IANBD, ThermoFisher) and three equivalents of TCEP to maintain reduced cysteine. Labeled BAX (BAX-NBD) was separated from unreacted IANBD by gel filtration (Econo-Pac 10 DG desalting column, BioRad) and used immediately. Translocation reactions were performed by combining 800 nM BAX with 1 µM BIM–BH3 or 200 nM tBID in the presence and absence of varying doses of EO. Reactions were initiated by the addition of 10 µL of the liposome stock. The NBD fluorophore exhibits low fluorescence in solution due to quenching by water. Upon BAX-NBD translocation, the NBD fluorophore is excluded from bulk water through contact with the liposomal membrane leading to an increase in fluorescence intensity. Fluorescence (*λ*ex = 475 nm and *λ*em = 530 nm) was measured at 1-min intervals at 37 °C indicated using a Tecan Infinite M1000 plate reader. The percentage translocation at any given time point was calculated as percentage translocation = [((F − F_0_)/(F_100_ − F_0_))(100)] − [((F_S_ − F_S0_)/(F_S100_ − F_S0_))(100)], where F_0_ and F_100_ are baseline and maximal fluorescence, respectively, and F_S_, F_S0_, and F_S100_ are the current fluorescence, baseline fluorescence, maximal fluorescence of solution BAX incubated in the absence of liposomes. The subtraction of the percent translocation of solution BAX is required to correct for NBD-fluorescence bleaching that occurs throughout the reaction. Triton X-100 (0.1%) was used to determine the maximum amount of liposomal translocation per assay and is set to F_100_ 100%.

### BAX conformation change assay using anti-6A7 immunoprecipitation

Exposure of the 6A7 epitope of BAX was assessed by immunoprecipitation with a 6A7-domain-specific antibody purchased from Santa Cruz (SC-23959). Protein G beads (50 µL, Santa Cruz) were washed three times with 3% BSA in PBS and incubated with 15 µL 6A7 antibody at 4 °C for 1 hr. Recombinant full-length BAX (10 µM) was incubated with four equivalents of BIM–BH3 peptide alone and in the presence of five or ten equivalents of EO for 15 min at room temperature. Incubation of full-length recombinant BAX with 0.1% Triton X served as a positive control for exposure of the 6A7 epitope. After incubation, 10 µL of each reaction was transferred to the protein G beads pre-loaded with anti-6A7 antibody and 1 µL was reserved as a loading control. After 90 min of incubation at 4 °C, beads were collected and washed three times with 500 µL of 3% BSA in PBS and solubilized with 25 µL LDS/DTT loading buffer. Samples were resolved by SDS-PAGE electrophoresis and western blot analysis with a BAX monoclonal antibody (Cell Signaling, Cat. # 2772) 1:1000.

### Western blotting and protein quantification

BAX samples were electrophoretically separated on 4–12% NuPage (Invitrogen) gels, transferred to mobilon-FL PVDF membranes (Millipore), and subjected to immunoblotting.(For visualization of proteins with Odyssey Infrared Imaging System (LI-COR Biosciences), membranes were blocked in PBS containing 2.5% milk powder. Primary BAX monoclonal antibody (Cell Signaling, Cat. #2772) was incubated overnight at 4 °C in a 1:1000 dilution. After washing, membranes were incubated with an IRdye800-conjugated goat anti-mouse IgG secondary antibody (LI-COR Biosciences, cat. # 925-32210) in a 1:5000 dilution. Protein was detected with Odyssey Infrared Imaging System. Densitometry of protein bands was acquired using an LI-COR Odyssey scanner. Quantification and analysis were performed using the western analysis tool from the Image Studio 3.1 software.

### NMR samples and spectroscopy

The uniformly ^15^N-labeled protein samples were prepared by growing the bacteria in a minimal medium, as previously described^44^. Unlabeled and ^15^N-labeled protein samples were prepared in 50 mM potassium phosphate, 50 mM NaCl solution at pH 6.0 in 10% D_2_O. All experiments were performed using an independent sample for each experimental measurement as a 400 μL sample in a 5-mm Shigemi; all samples were DMSO matched with 2% d_6_-DMSO. Correlation ^1^H-^15^N-HSQC spectra were recorded on ^15^N-labeled BAX at 50 μM in the presence and absence of 100 μM of EO. NMR spectra were acquired at 25 °C on a Bruker 600 MHz spectrometer equipped with a cryoprobe, processed using TopSpin and analyzed using NMRView. BAX cross-peak assignments were applied as previously reported^17^. The weighted average chemical shift perturbation (CSP) was calculated as √(Δδ^1^H)^2^ + (Δδ^15^N/5)^2^)/2 in p.p.m. The absence of a bar indicates no chemical shift difference, the presence of proline, or a residue that is overlapped or missing and therefore not used in the analysis. The significance threshold for backbone amide chemical shift changes was calculated based on the average chemical shift across all residues plus 0.5 or 1 s.d^48^. The solvent-accessible surface area was probed by the addition of 10 mM hy-TEMPO (Sigma) to 50 µM ^15^N-labeled BAX with and without 100 µM EO measured using standard ^1^H-^15^N-HSQC with an increased recycle delay of 2 s^30,49^ PRE was calculated as the ratio of peak intensities of BAX in the presence of hy-TEMPO to BAX without hy-TEMPO (% intensity). Mapping of chemical shifts and PRE data onto the BAX structure was performed with PyMOL (Schrodinger, LLC, 2018–2019). The software was made available through the SBGrid collaborative network^50^.

### NMR-based docking calculations and molecular dynamics

NMR-guided docking of EO into the NMR structure of BAX (PDB: 1F16: 10.2210/pdb1F16/pdb) was performed using induced-fit docking (IFD, Schrodinger, LLC, 2018) with extra precision (XP) and a binding site at the mid-point of residues K21, R134, and R145. EO was converted to 3D all atom structure using LIGPREP (Schrodinger, LLC, 2018) and assigned partial charges with EPIK (Schrodinger, LLC, 2018). Poses generated were consistent with NMR data and indicated a strong favoring of ionic interaction between the carboxylate of EO and a basic residue of BAX. Mutagenesis was used to elucidate the pose of EO on the trigger site of BAX. The pose consistent with mutant BAX liposomal release data was most consistent with NMR CSP data. This pose was subjected to three independent 100 nsec molecular dynamics (MD) simulations using DESMOND (DESMOND, version 3, Schrodinger, LLC, 2017). Three independent 100 ns MD simulations were also performed with the lowest energy BAX structure from the NMR ensemble (PDB 1F16: 10.2210/pdb1F16/pdb). MD runs were performed in a truncated octahedron SPC water box using OPLS_2005 force field, 300 K, and the constant pressure of 1.0325 bar. Analysis of the trajectory was performed with MAESTRO simulation event analysis tools (Schrodinger, LLC, 2018). PyMOL (Schrodinger, LLC, 2018–2019) was used for preparing the highlighted poses. The %ΔRMSF for each residue was calculated as %ΔRMSF = ((RMSF_EO_ – RMSF_Apo_)100/RMSF_Apo_), where RMSF_EO_ was the RMSF of an individual MD simulation of EO docked into BAX and RMSF_Apo_ is the average RMSF of the apo BAX simulation. Distance frequency histograms were prepared using GraphPad Prism frequency distribution analysis. The docked binding poses of BAM7, BTSA1, and EO within the BAX trigger site were used to calculate the binding energy of each compound using the MM-GBSA module^51^ in Maestro (Schrödinger, LLC, NY, 2018–2019). Docked complexes were analyzed with the MM-GBSA module using VSGB solvation model and OPLS3 force field.

### Structural analysis

Structural analysis was performed in PyMOL (Schrödinger, LLC: NY, 2018–2019) and Maestro tools SiteMap, GLIDE, EPIC, and LIGPREP (Schrödinger, LLC, NY, 2018–2019).

### Cytochrome *c* release assay

*BAX*^−/−^ or *BAK*^−/−^ mouse embryonic fibroblasts were maintained in DMEM (Life Technologies) supplemented with 10% FBS, 100 U/mL penicillin/streptomycin, 2 mM l-glutamine, and 0.1 mM MEM nonessential amino acids. MEFs (5 × 10^4^ cells/well) were seeded in a 96-well clear u-bottom plate for 18–24 h. BAX or BAK expression was confirmed with immunoblot (anti-BAX antibody 2772S, Cell Signaling, 1:1000 dilution and anti-BAK antibody 06-536, Millipore, 1:1000 dilution). Media was removed and replaced with media lacking FBS, and cells were treated with varying doses of EO for 2 h at 37 °C. After incubation, the media was removed and replaced with 100 µL reaction buffer modified from MEB buffer (150 mM mannitol, 10 mM HEPES-KOH pH 7.5, 50 mM KCl, 0.02 mM EGTA, 0.02 mM EDTA, 0.1% BSA, 5 mM succinate, 20 µg/mL oligomycin, 10 mM DTT, and 0.00125% digitonin) with and without 5 µM BIM–BH3 peptide and incubated at 30 °C for 45 min. After incubation, an additional 100 µL of reaction buffer was added and the plate was gently tapped to mix. Cytochrome *c* release was determined by decanting 50 µL of the supernatant and analyzing with the rat/mouse cytochrome *c* quantikine ELISA kit (R&D Systems, MCT0) according to the recommended protocol. Percentage inhibition was normalized to BIM–BH3 peptide alone (0%) and untreated cells (100%).

### In cell BAX translocation assays

BAK KO Cells (1 × 10^6^ cells/well) were seeded in 10-cm clear bottom plate overnight in the media described above. The media was removed and replaced with media lacking FBS. Cells were then pre-treated with 10 or 20 µM EO as a 10X stock in dH_2_O or dH_2_O vehicle for 1 h. After incubation, the media was removed, cells were washed with PBS, harvested and resuspended in 100 µL reaction buffer modified from MEB buffer (with and without 10 µM and 20 µM BIM–BH3 peptide and incubated at 30 °C for 1 h. Reactions were then moved to ice and supplemented with an additional 0.025% digitonin for 10 min. The cytosolic fraction was isolated by centrifugation for 10 min at 1500 × *g*. The mitochondrial pellet was solubilized using PBS + 1% Triton X 100 for 1 hr followed by centrifugation at 21,000 × *g*. Protein concentrations were normalized and resolved by SDS-PAGE electrophoresis and western blot analysis using 4–12% NuPage (Life Technologies) gels, and analyzed by immunoblotting with anti-BAX antibody (2772S, Cell Signaling, 1:1000 dilution). VDAC1 (Cell Signaling, Cat. # 4661, 1:1000 dilution) and b-Actin (Sigma, Cat. A1978, 1:5000 dilution) are used for loading control of mitochondrial and supernatant fractions respectively. For detection of BAX translocation using confocal microscopy, BAK KO MEFs were treated with 10 μΜ EO at ∼70% confluence in media lacking FBS for 2 h prior to the addition of 10% FBS then and 2 μM of STS or DMSO for 4.5 h. After treatments, cells were washed with PBS and fixed with 4% PFA for 15 min. Then cells were permeabilized with 0.1% Triton X-100 in PBS for 10 min. Cells were blocked with 5% BSA in PBS-T and incubated overnight with primary antibodies as indicated; BAX (Cell Signaling Technology; 2772S, 1:100 dilution) and TOMM20 (Sigma; ST1705, 1:100 dilution). After incubation with primary antibodies, cells were washed with PBS and incubated with the appropriate mouse or rabbit secondary antibodies (ThermoFisher Scientific; A11008, A11030, 1:500 dilution) in the blocking solution. After PBS washes, coverslips were dipped in water and mounted on glass slides using Vectashield containing DAPI (Vector laboratories). Images were taken with Leica SP5 inverted confocal microscope. Data were analyzed with ImageJ.

### Cell viability and caspase-3/7 activation assays

3T3 cells were maintained in media identical to that of MEFs. 3T3 cells were seeded (1 × 10^4^ cells/well) in 96-well opaque plates for 18–24 h. The media was removed and replaced with media lacking FBS and cells were treated with EO as a 10× stock in H_2_O at the indicated doses for 2 h before addition of 10% FBS. Cells were then treated with 1 µM each of ABT-263 and S63845. Caspase-3/7 activation was measured at 4 h by addition of the Caspase-Glo 3/7 chemiluminescence reagent in accordance with the manufacturer’s protocol (Promega). Luminescence was detected by an F200 PRO microplate reader (TECAN). Percentage caspase activation was normalized to ABT-263 + S63845 alone (100%) and untreated cells (0%). Viability assays were performed at 24 h by addition of CellTiter-Glo according to the manufacturer’s protocol (Promega). Luminescence was detected by an F200 PRO microplate reader (TECAN). Percentage viability was normalized to untreated cells (100%). For caspase-3/7 assay in BAK KO and BAX KO MEFs, cells (2.5 × 10^3^ cells/well) were seeded in a 384-well white plate and treated with EO as a 10× stock in DMSO at the indicated doses in the absense of FBS for 2 h prior to staurosporine treatment and addition of 10% FBS. Caspase-3/7 activation was measured at 6 h by addition of the Caspase-Glo 3/7 chemiluminescence reagent in accordance with the manufacturer’s protocol (Promega). Luminescence was detected by a F200 PRO microplate reader (TECAN). Caspase assays were performed in at least triplicate and the data normalized to cell death stimulus-treated wells. Dilutions of EO or staurosporine were performed using a TECAN D300e Digital Dispenser from DMSO stocks.

### Cellular thermal shift assays (CETSA)

BAK KO MEFs were seeded in a 10-cm dish for 18–24 h or until ~80% confluent. The media was removed and replaced with media lacking FBS and cells were treated with 10 µM EO as a 10× stock in H_2_O or vehicle for 2 h. The media was then removed and cells were harvested using a cell scraper and washed twice with PBS. Cells were then resuspended in PBS to 6 × 10^6^ cells/mL and 50 µL was transferred to PCR tubes. Cells were then heated in a Biorad C1000 Touch Thermal Cycler for 3 min using a temperature gradient (50, 52.1, 55.4, 59.4, 64.9, 69.2, 72.1, and 74 °C). Cells remaining at room temperature (25 °C) served as a control. All cells were lysed by three cycles of freeze-thawing using liquid nitrogen. Samples were then centrifuged at 2 × 10^4^ × *g* for 15 min. The supernatants were collected and resolved by SDS-PAGE with an N-terminal BAX antibody (Cell Signaling, 2772S). Samples were analyzed and quantified using a Li-Cor Odyssey Clx and normalized to 25 °C (100%) and 74 °C (0%).

### Calculation of recombinant BAX T_M_

Purified recombinant BAX (25 µM) was combined with DMSO or EO (1:10 BAX:EO) and loaded into Tycho NT.6 (NanoTemper Technologies) capillaries. First derivative (330/350 nm) melting point curves were generated automatically using the Tycho NT.6 (NanoTemper Technologies). Data were exported and to GraphPad PRISM software for analysis and visualization.

### Statistical analysis

Statistical significance for pair-wise comparison of groups was determined by two-tailed Student’s *t* test using GraphPad PRISM software (GraphPad Inc., CA). *P* values of less than 0.05 were considered significant.

### Reporting summary

Further information on research design is available in the Nature Research Reporting Summary linked to this article.

## Supplementary information

Supplementary Information

Peer Review

Reporting Summary

## Data Availability

Data generated or analyzed during this study are included in this published article and its Supplementary Information files and are available from the corresponding author on a reasonable request. The following publicly available datasets were used in the production of this paper: PDB 1F16 [10.2210/pdb1F16/pdb], PDB 2K7W [10.2210/pdb2K7W/pdb]. Source data are provided with this paper.

## Source data

Source Data
