# Supplementary material for: Eltrombopag directly inhibits BAX and prevents cell death
Source: Nat Commun. 2021 Feb 18;12:1134. doi: 10.1038/s41467-021-21224-1 (PMC7892824; doi:10.1038/s41467-021-21224-1)
Supplement: Supplementary file 1 — Supplementary Information [file 41467_2021_21224_MOESM1_ESM.pdf]

Supplementary Information

Eltrombopag directly inhibits BAX and prevents cell death

Spitz et al.

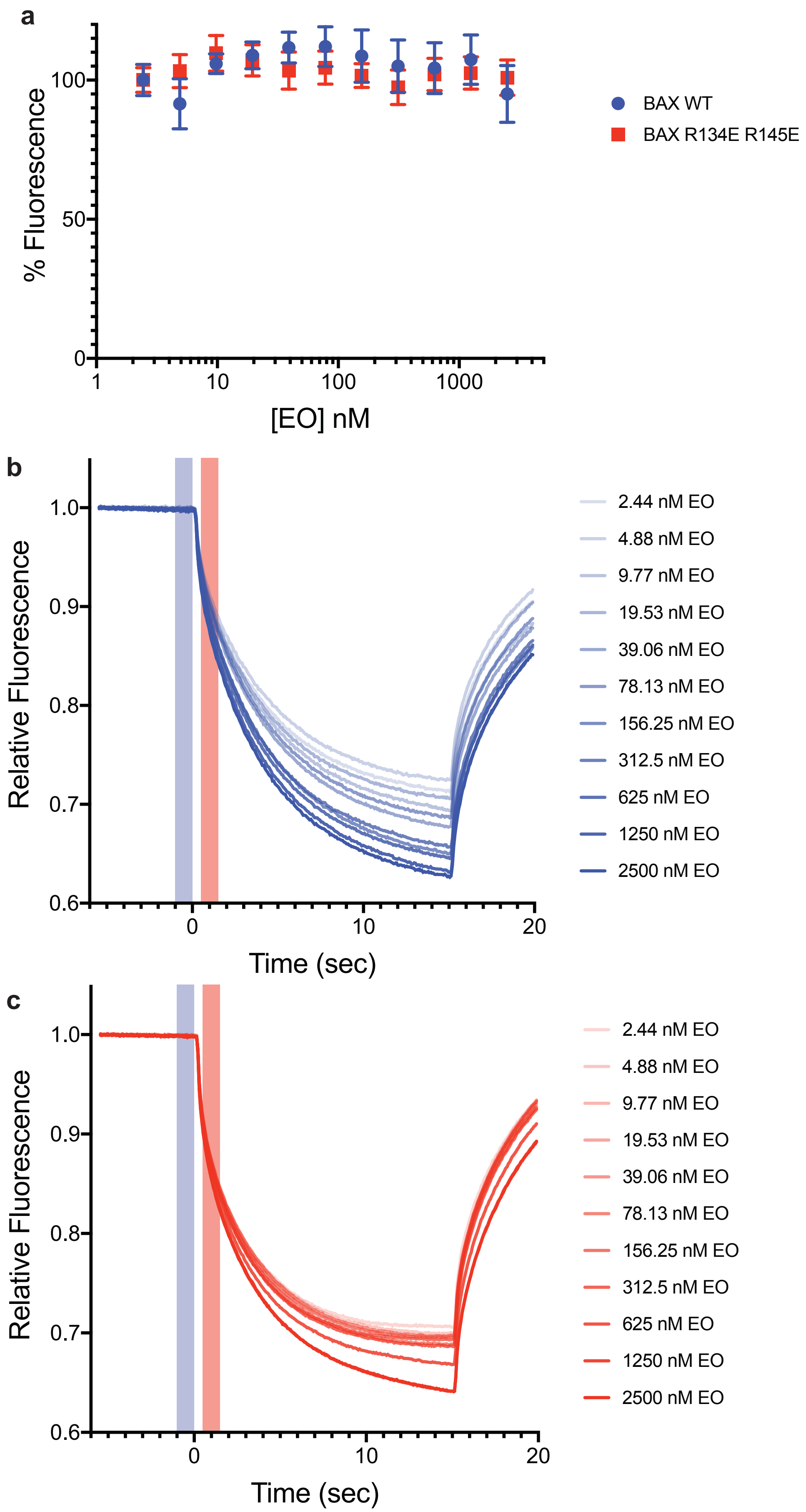
a
% Fluorescence
100
50
0
1
10
100
1000
[EO] nM
BAX WT
BAX R134E R145E
b
Relative Fluorescence
1.0
0.9
0.8
0.7
0.6
0
10
20
Time (sec)
2.44 nM EO
4.88 nM EO
9.77 nM EO
19.53 nM EO
39.06 nM EO
78.13 nM EO
156.25 nM EO
312.5 nM EO
625 nM EO
1250 nM EO
2500 nM EO
c
Relative Fluorescence
1.0
0.9
0.8
0.7
0.6
0
10
20
Time (sec)
2.44 nM EO
4.88 nM EO
9.77 nM EO
19.53 nM EO
39.06 nM EO
78.13 nM EO
156.25 nM EO
312.5 nM EO
625 nM EO
1250 nM EO
2500 nM EO

Supplementary Figure 1. Direct binding of Eltrombopag (EO) to BAX by microscale thermophoresis (MST). a, Normalized percent fluorescence of BAX 4C and BAX 4C R134E R145E upon titration of EO over a range of concentrations showing stable fluorescence. **b-c,** MST thermophoretic traces of fluorescence with respect to time for BAX-4C + EO (**b**) and BAX-4C R134E R145E + EO (**c**). MST titration curves were determined using the temperature jump portion of the curve (red shaded box) normalized to the pre-IR region (blue shaded box). Data are representative of three independent experiments each n=3 ± SEM. Source data has been provided.

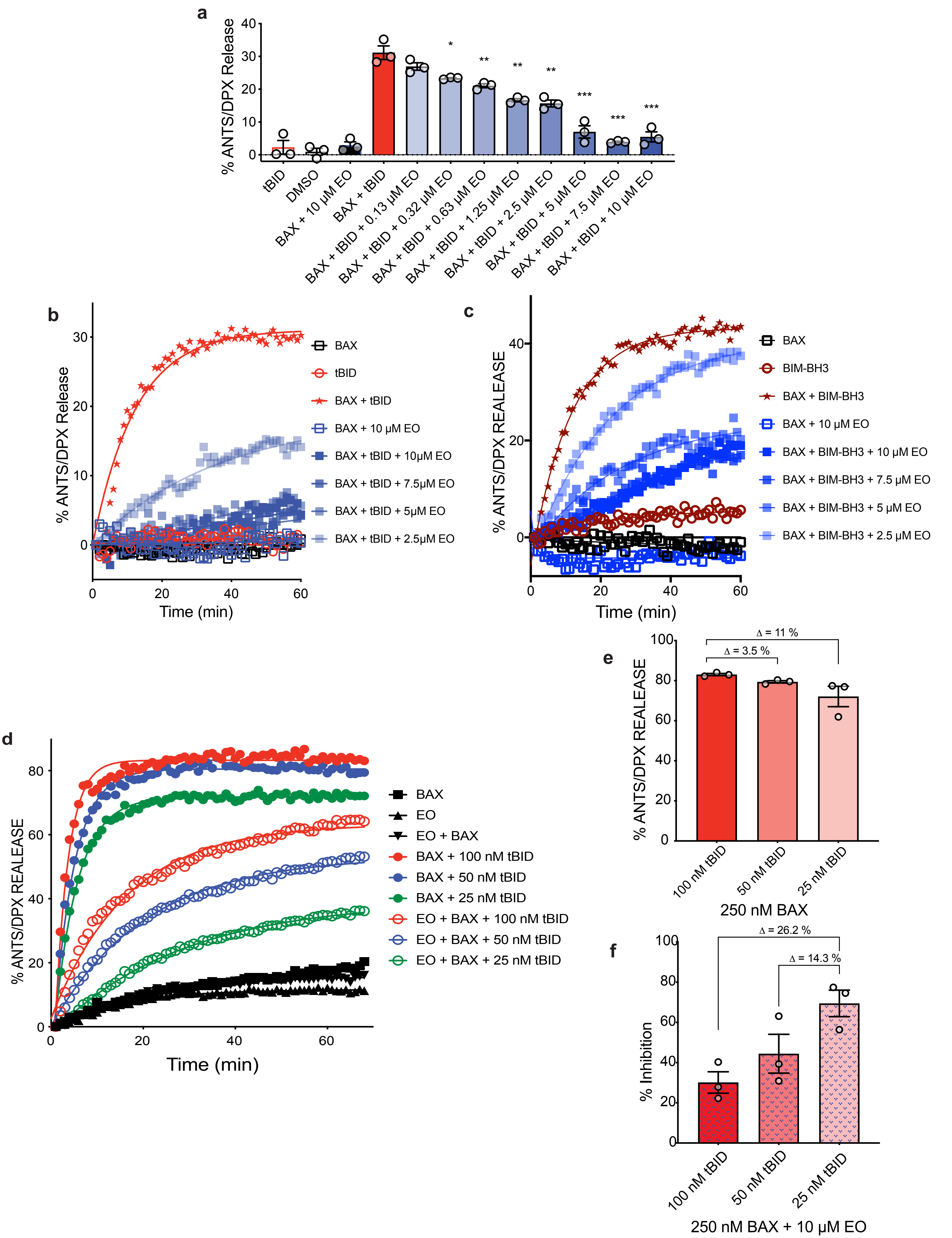

a
% ANTS/DPX Release
40
30
20
10
0
*
**
**
**
***
***
***
tBID
DMSO
BAX + 10 μM EO
BAX + tBID
BAX + tBID + 0.13 μM EO
BAX + tBID + 0.32 μM EO
BAX + tBID + 0.63 μM EO
BAX + tBID + 1.25 μM EO
BAX + tBID + 2.5 μM EO
BAX + tBID + 5 μM EO
BAX + tBID + 7.5 μM EO
BAX + tBID + 10 μM EO
b
% ANTS/DPX Release
Time (min)
BAX
tBID
BAX + tBID
BAX + 10 μM EO
BAX + tBID + 10μM EO
BAX + tBID + 7.5μM EO
BAX + tBID + 5μM EO
BAX + tBID + 2.5μM EO
c
% ANTS/DPX REALEASE
Time (min)
BAX
BIM-BH3
BAX + BIM-BH3
BAX + 10 μM EO
BAX + BIM-BH3 + 10 μM EO
BAX + BIM-BH3 + 7.5 μM EO
BAX + BIM-BH3 + 5 μM EO
BAX + BIM-BH3 + 2.5 μM EO
d
% ANTS/DPX REALEASE
Time (min)
BAX
EO
EO + BAX
BAX + 100 nM tBID
BAX + 50 nM tBID
BAX + 25 nM tBID
EO + BAX + 100 nM tBID
EO + BAX + 50 nM tBID
EO + BAX + 25 nM tBID
e
% ANTS/DPX REALEASE
Δ = 11 %
Δ = 3.5 %
100 nM tBID
50 nM tBID
25 nM tBID
250 nM BAX
f
% Inhibition
Δ = 26.2 %
Δ = 14.3 %
100 nM tBID
50 nM tBID
25 nM tBID
250 nM BAX + 10 μM EO

Supplementary Figure 2. Eltrombopag (EO) inhibits BH3-induced BAX activation. **a**, Representative BAX-mediated membrane permeabilization assay using ANTS/DPX liposomes with 50 nM BAX, 5 nM tBID, and 0.13-10 μM EO after 30 minute incubation. Data are representative of three independent experiments each n=3 ± SEM. **b**-**c**, Representative kinetic traces of ANTS/DPX liposomal release with respect to time in the presence and absence of 2.5-10 μM EO. Conditions are 50 nM BAX + 5 nM tBID (**b**) and 50 nM BAX + 1 μM BIM-BH3 (**c**). **d**, Kinetic traces of ANTS/DPX liposomal release with respect to time in the presence of 250 nM BAX, 10 μM EO, and 25, 50, or 100 nM tBID. **e**-**f**, Quantified ANTS/DPX liposomal release after 60 minutes for BAX in the presence of the indicated concentration of tBID (**e**) and corresponding percentage inhibition in the presence of 10 μM EO (**f**). Data are representative of three independent experiments each n=3 ± SEM. Two-sided *t*-test, **** $P < 0.0001$; ***$P < 0.001$;**$P < 0.01$; *$P < 0.05$; ns, $P > 0.05$ (Left to right P-values were 0.0204, 0.0097, 0.0025, 0.0027, 0.0010, 0.0002, 0.0006 for (**a**)). Source data has been provided.

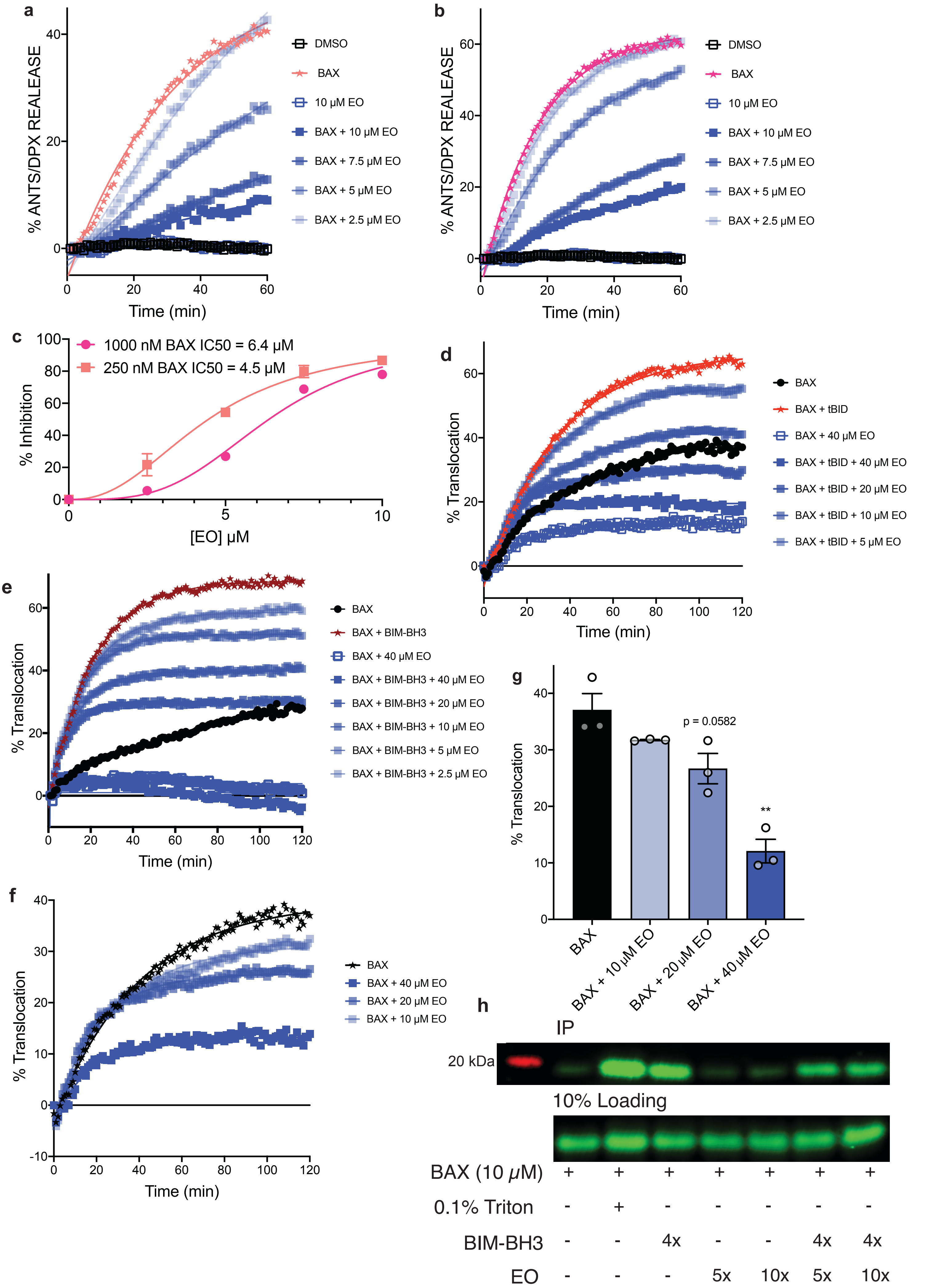
a
% ANTS/DPX REALEASE
Time (min)
DMSO
BAX
10 µM EO
BAX + 10 µM EO
BAX + 7.5 µM EO
BAX + 5 µM EO
BAX + 2.5 µM EO
b
% ANTS/DPX REALEASE
Time (min)
DMSO
BAX
10 µM EO
BAX + 10 µM EO
BAX + 7.5 µM EO
BAX + 5 µM EO
BAX + 2.5 µM EO
c
1000 nM BAX IC50 = 6.4 µM
250 nM BAX IC50 = 4.5 µM
% Inhibition
[EO] µM
d
% Translocation
Time (min)
BAX
BAX + tBID
BAX + 40 µM EO
BAX + tBID + 40 µM EO
BAX + tBID + 20 µM EO
BAX + tBID + 10 µM EO
BAX + tBID + 5 µM EO
e
% Translocation
Time (min)
BAX
BAX + BIM-BH3
BAX + 40 µM EO
BAX + BIM-BH3 + 40 µM EO
BAX + BIM-BH3 + 20 µM EO
BAX + BIM-BH3 + 10 µM EO
BAX + BIM-BH3 + 5 µM EO
BAX + BIM-BH3 + 2.5 µM EO
f
% Translocation
Time (min)
BAX
BAX + 40 µM EO
BAX + 20 µM EO
BAX + 10 µM EO
g
% Translocation
p = 0.0582
**
BAX
BAX + 10 µM EO
BAX + 20 µM EO
BAX + 40 µM EO
h
IP
20 kDa
10% Loading
BAX (10 µM) + + + + + + +
0.1% Triton - + - - - - -
BIM-BH3 - - 4x - - 4x 4x
EO - - - 5x 10x 5x 10x

Supplementary Figure 3. Eltrombopag (EO) inhibits heat-induced BAX activation, translocation and 6A7-epitope exposure. a-b, Representative kinetic traces of ANTS/DPX liposomal release with respect to time in the presence and absence of 2.5-10 μM EO. Conditions are 250 nM BAX (**a**) or 1000 nM BAX (**b**) at 42°C. **c**, Percentage inhibition dose response curves for EO inhibition of heat induced BAX activation at 250 and 1000 nM at 30 minutes incubation. IC_{50} values are shown for clarity. **d-f**, Representative kinetic traces of NBD-labeled BAX (800 nM) translocation with respect to time in the presence of 200 nM tBID (**d**), 1 μM BIM-BH3 (**e**), and 37°C heat (**f**) at the indicated concentration of EO up to 40 μM. **g**, Quantified percentage auto-translocation of NBD-labeled BAX (800 nM) at 37°C in the presence of 10-40 μM EO. Data are representative of two independent experiments each n=3 ± SEM. Two-sided *t*-test, **** *P* < 0.0001; ****P* < 0.001;***P* < 0.01; **P* < 0.05; ns, *P* > 0.05 (Left to right P-values were 0.0582, 0.0022 for (**g**)). **h**, BIM-BH3 mediated BAX activation immunoprecipitation assay using the 6A7-epitope specific antibody with 1:4 BAX:BIM-BH3 and 1:5 or 1:10 BAX:EO. Immunoblot is representative of two independent experiments. Source data has been provided.

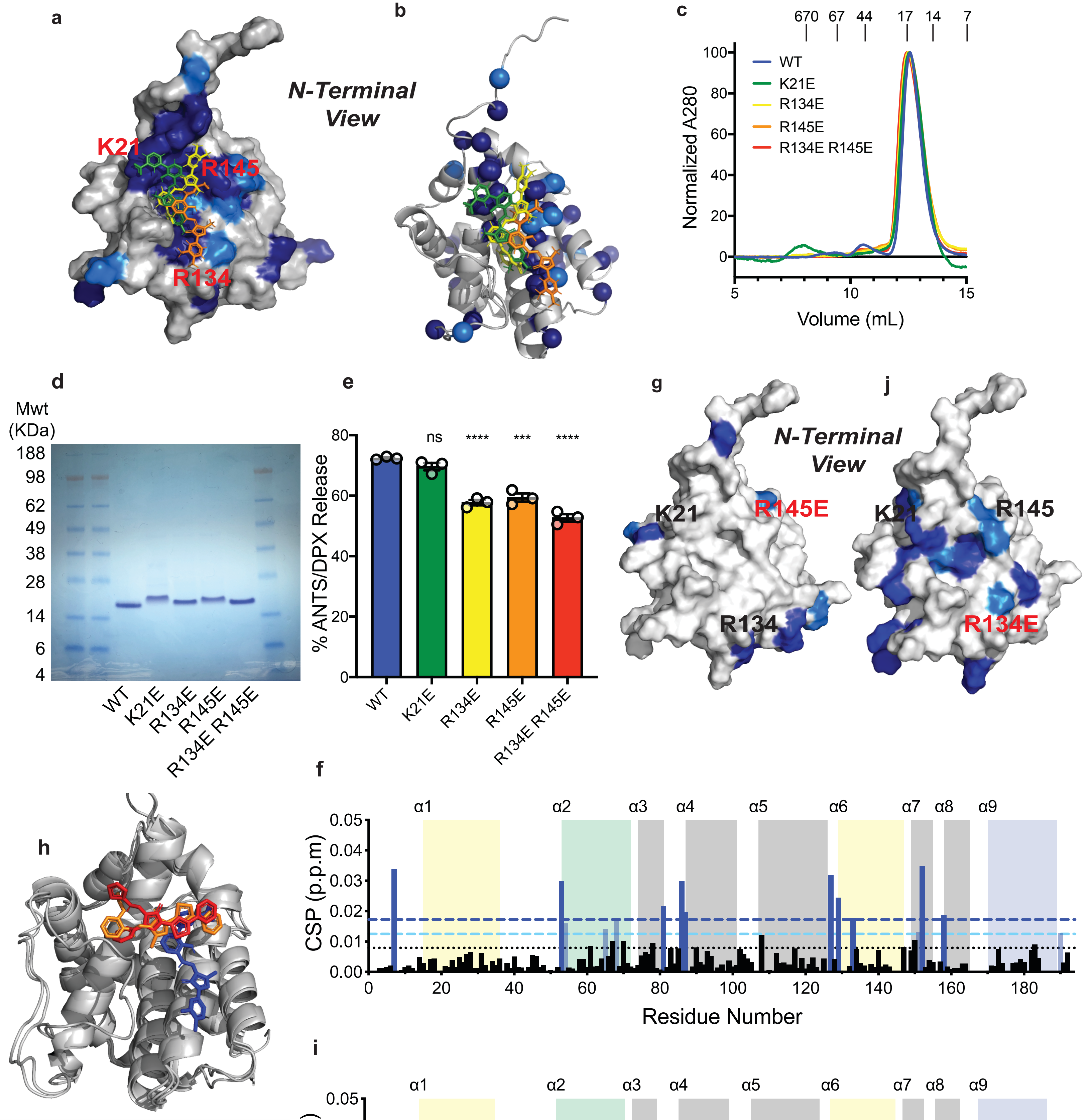

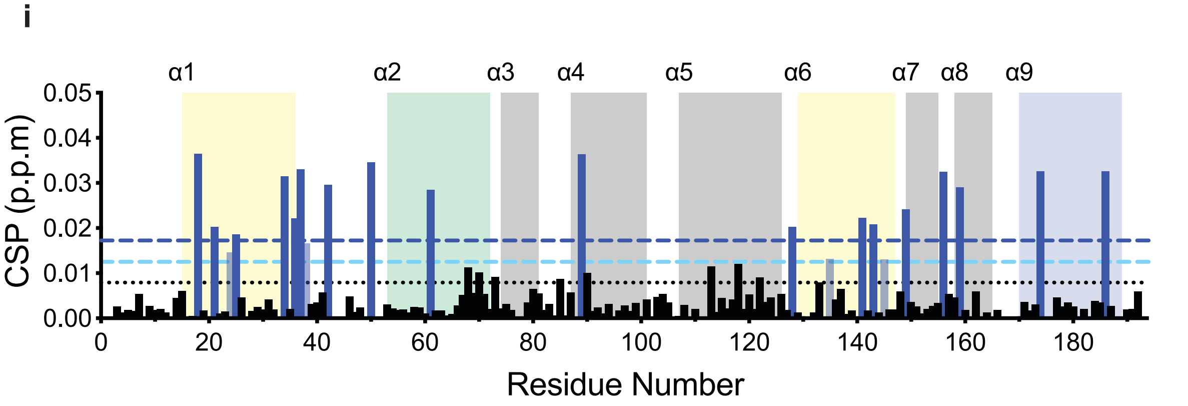

Ligand	FPA IC50 (nM)	MM-GBSA Binding Energy (kcal/mol)
BAM7	3310	-46.8
BTSA1	250	-48.6
EO	207	-53.2

Supplementary Figure 4. Eltrombopag (EO) forms unique contacts with the BAX trigger site. a-b, Surface (**a**) and ribbon (**b**) representation of HSQC-NMR restraint guided induced fit docking (IFD) of EO onto the N-terminal trigger site of BAX (PDB: 1F16 DOI: 10.2210/pdb1F16/pdb). Sample poses representative of ionic interactions between EO and one of three basic trigger site residues are displayed in different colors for clarity. Exact glide docking scores are as follows: orange (R145), -6.111; yellow (R134), -6.881; green (K21), -4.687. Data are representative of three independent experiments. **c,** Size exclusion chromatography traces using Superdex 75 Increase FPLC column of wild type (WT) BAX and the trigger site mutants K21E, R134E, R145E, and R134E R145E. All mutants elute as monomers with no significant differences in elution profile. **d,** SDS-PAGE of WT BAX and the trigger site mutants K21E, R134E, R145E, and R134E R145E showing a consistent ~21 kDa band of acceptable purity. **e,** Percentage ANTS/DPX liposomal release of 250 nM WT (blue), K21E (green), R134E (yellow), R145E (orange) or R134E R145E (red) BAX and 5 nM tBID after 30 minutes. Data are representative of two independent experiments each n=3 ± SEM. Two- sided *t*-test, **** $P < 0.0001$; ***$P < 0.001$;**$P < 0.01$; *$P < 0.05$; ns, $P > 0.05$ (Left to right P-values were 0.00009, 0.0007, 0.00009 for (**e**)). **f,** Measured chemical shift perturbations (CSPs) of ^{15}N-labeled BAX R145E in the presence of 1:2 BAX R145E:EO are plotted as a function of BAX residue number. Residues with chemical shift perturbations over the significance threshold or 2 times the significance threshold are labeled light blue or dark blue, respectively. The black dotted line represents the average CSP. Residues associated with the N-terminal trigger site, BH3-domain, canonical site, and transmembrane domain are highlighted in yellow, green, grey, and blue, respectively. Data is representative of three independent experiments. **g**, Mapping of significant CSPs noted in BAX R145E (**f**) to the surface structure of BAX (PDB: 1F16 DOI: 10.2210/pdb1F16/pdb). **h**, Overlay of BAX docking poses for EO, BAM7, and BTSA1 showing different trigger site binding orientations. Previously reported fluorescence polarization competition assay IC50 (FPA IC50) correlates with binding energy predicted by Schrodinger Maestro MM-GBSA. **i,** Measured chemical shift perturbations (CSPs) of ^{15}N-labeled BAX R134E in the presence of 1:2 BAX R145E:EO are plotted as a function of BAX residue number. Colors are as described for **f**. **j**, Mapping of significant CSPs noted in BAX R134E (**i**) to the surface structure of BAX (PDB: 1F16 DOI: 10.2210/pdb1F16/pdb). Source data has been provided.

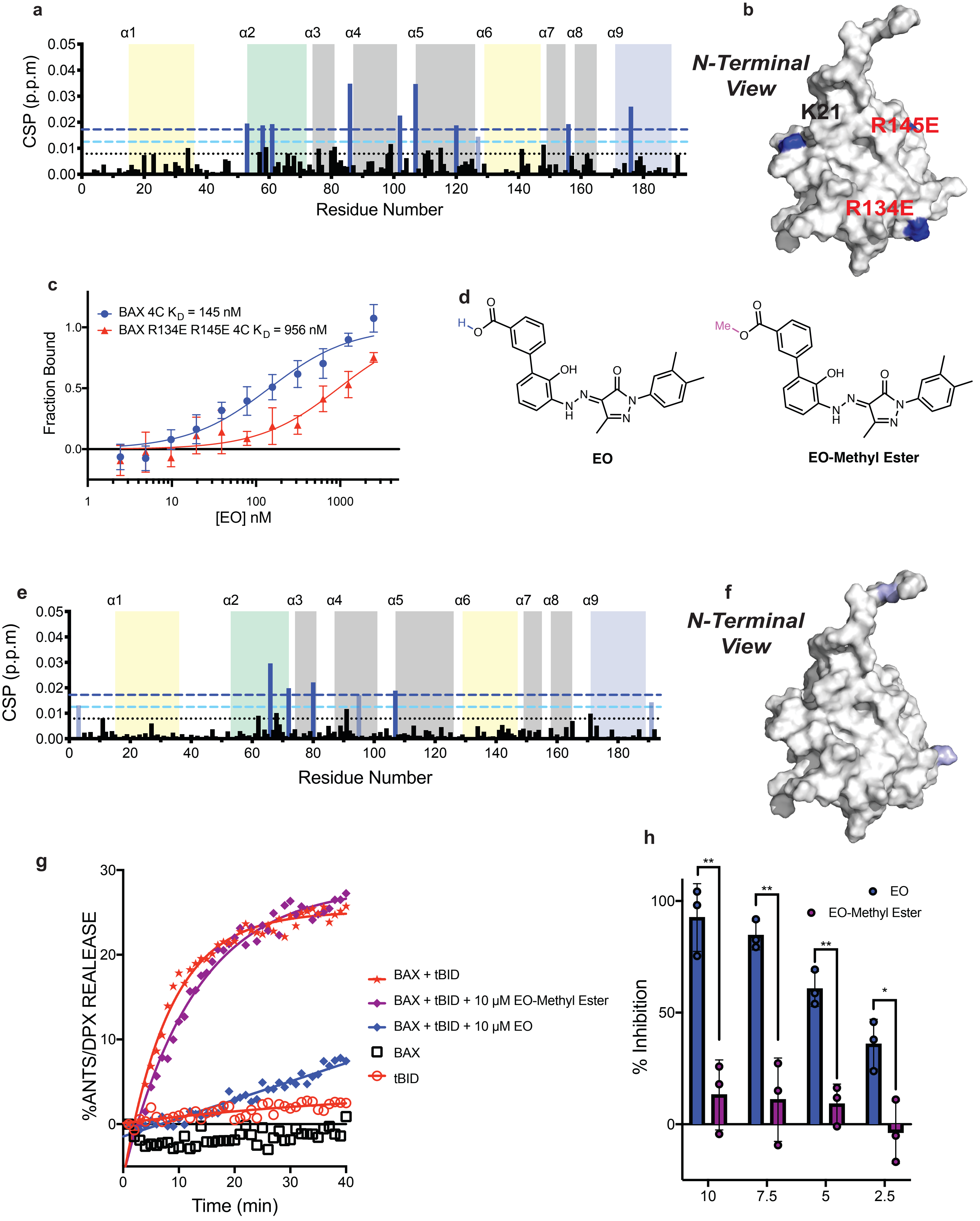

a
α1
α2
α3
α4
α5
α6
α7
α8
α9
CSP (p.p.m)
Residue Number
b
N-Terminal View
K21
R145E
R134E
c
BAX 4C K_D = 145 nM
BAX R134E R145E 4C K_D = 956 nM
Fraction Bound
[EO] nM
d
EO
EO-Methyl Ester
e
α1
α2
α3
α4
α5
α6
α7
α8
α9
CSP (p.p.m)
Residue Number
f
N-Terminal View
g
%ANTS/DPX REALEASE
Time (min)
BAX + tBID
BAX + tBID + 10 μM EO-Methyl Ester
BAX + tBID + 10 μM EO
BAX
tBID
h
% Inhibition
EO
EO-Methyl Ester

Supplementary Figure 5. Eltrombopag (EO) binding requires a critical ionic interaction with BAX R145. a, Measured chemical shift perturbations (CSPs) of ^{15}N-labeled BAX R134E R145E in the presence of 1:2 BAX R134E R145E:EO are plotted as a function of BAX residue number. Residues with chemical shift perturbations over the significance threshold or 2 times the significance threshold are labeled light blue or dark blue, respectively. The black dotted line represents the average CSP. Residues associated with the N-terminal trigger site, BH3-domain, canonical site, and transmembrane domain are highlighted in yellow, green, grey, and blue, respectively. Data is representative of three independent experiments. **b,** Mapping of significant CSPs noted in (**a)** to the surface (left) structure of BAX (PDB: 1F16 DOI: 10.2210/pdb1F16/pdb). **c,** Microscale thermophoresis (MST) direct binding of EO to wild type BAX- 4C and BAX-4C R134E R145E. Data are representative of two independent experiments each n=3 ± SEM. K_D is shown for clarity. **d,** Chemical structures of EO and EO-methyl-ester. **e,** Measured chemical shift perturbations (CSPs) of ^{15}N-labeled BAX in the presence of 1:2 BAX:EO-methyl ester are plotted as a function of BAX residue number. Data is representative of three independent experiments. **f,** Mapping of significant CSPs noted in (e) to the surface (left) structure of BAX (PDB: 1F16 DOI: 10.2210/pdb1F16/pdb). g, Representative kinetic traces of ANTS/DPX liposomal release with respect to time in the presence of 10 μM EO or EO-methyl ester. Conditions are 50 nM BAX and 5 nM tBID. Data are representative of two independent experiments each n=3 ± SEM. h, Percentage inhibition of tBID induced BAX mediated liposomal permeabilization by EO and EO-methyl ester. Data are representative of two independent experiments each n=3 ± SEM. Two-sided t-test, **** P < 0.0001; ***P < 0.001;**P < 0.01; *P < 0.05; ns, P > 0.05 (Left to right P-values were 0.0032, 0.0029, 0.0017, 0.0188 (h)). Source data has been provided.

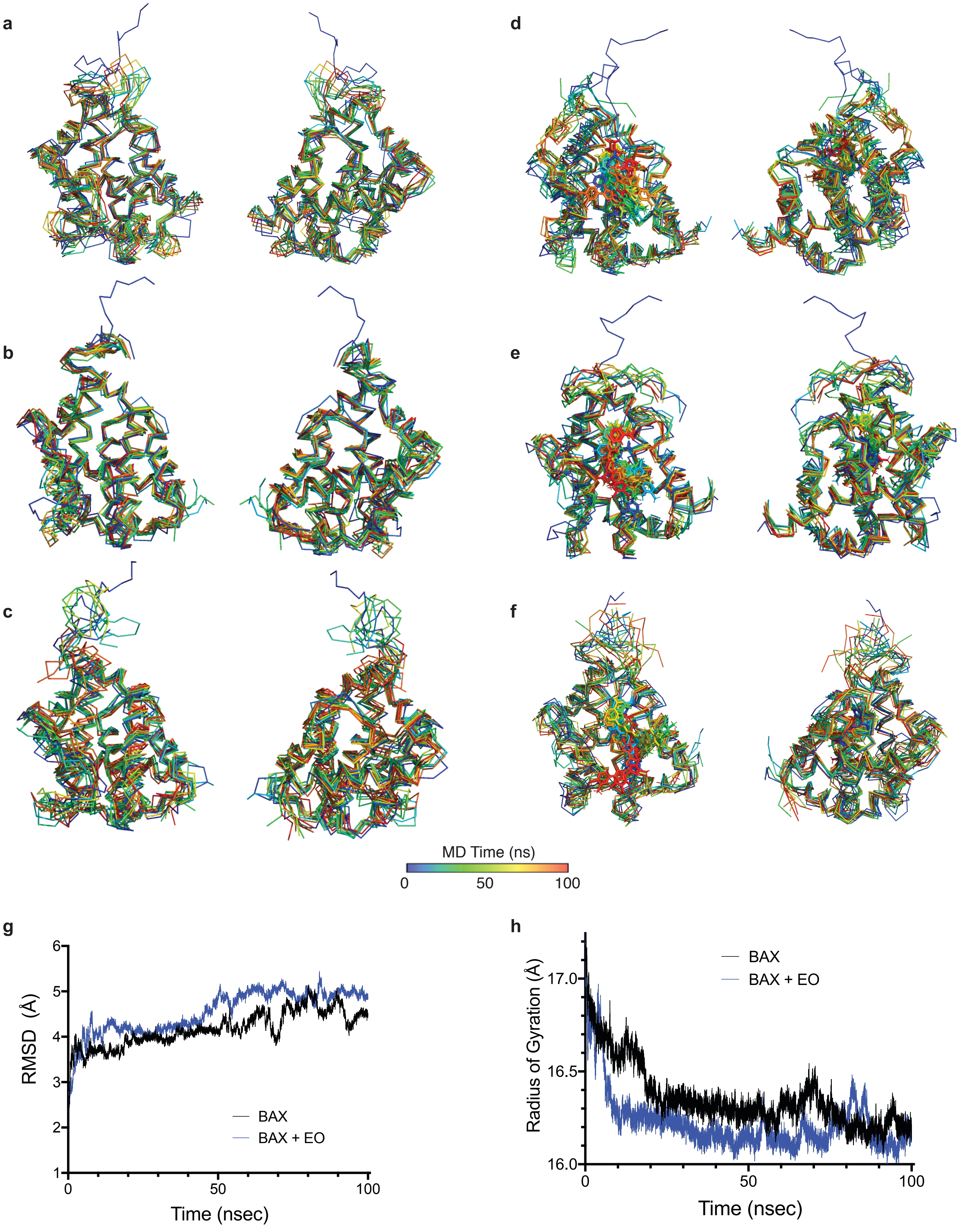

a
b
c
d
e
f
MD Time (ns)
0
50
100
g
RMSD (Å)
Time (nsec)
BAX
BAX + EO
h
Radius of Gyration (Å)
Time (nsec)
BAX
BAX + EO

Supplementary Figure 6. Molecular dynamics (MD) simulations of unbound BAX and the BAX:EO complex. a-f, Overlay of structures from 100 nsec MD simulation of BAX (**a-c**) and BAX:EO (**d-f**) in 10 nsec intervals as indicated by the color scale. N-terminal (left) and C-terminal (right) views are shown for clarity. Three independent simulations were performed (**a-c** and **d-f**). **g,** Root mean square deviation (RMSD) of atomic positions is plotted with respect to time for BAX and BAX-EO. Mean RMSD from n=3 BAX and BAX-EO simulations. **h,** Radius of gyration plotted with respect to time for BAX and BAX-EO. Mean radius of gyration from n=3 BAX and BAX-EO simulations. Source data has been provided.

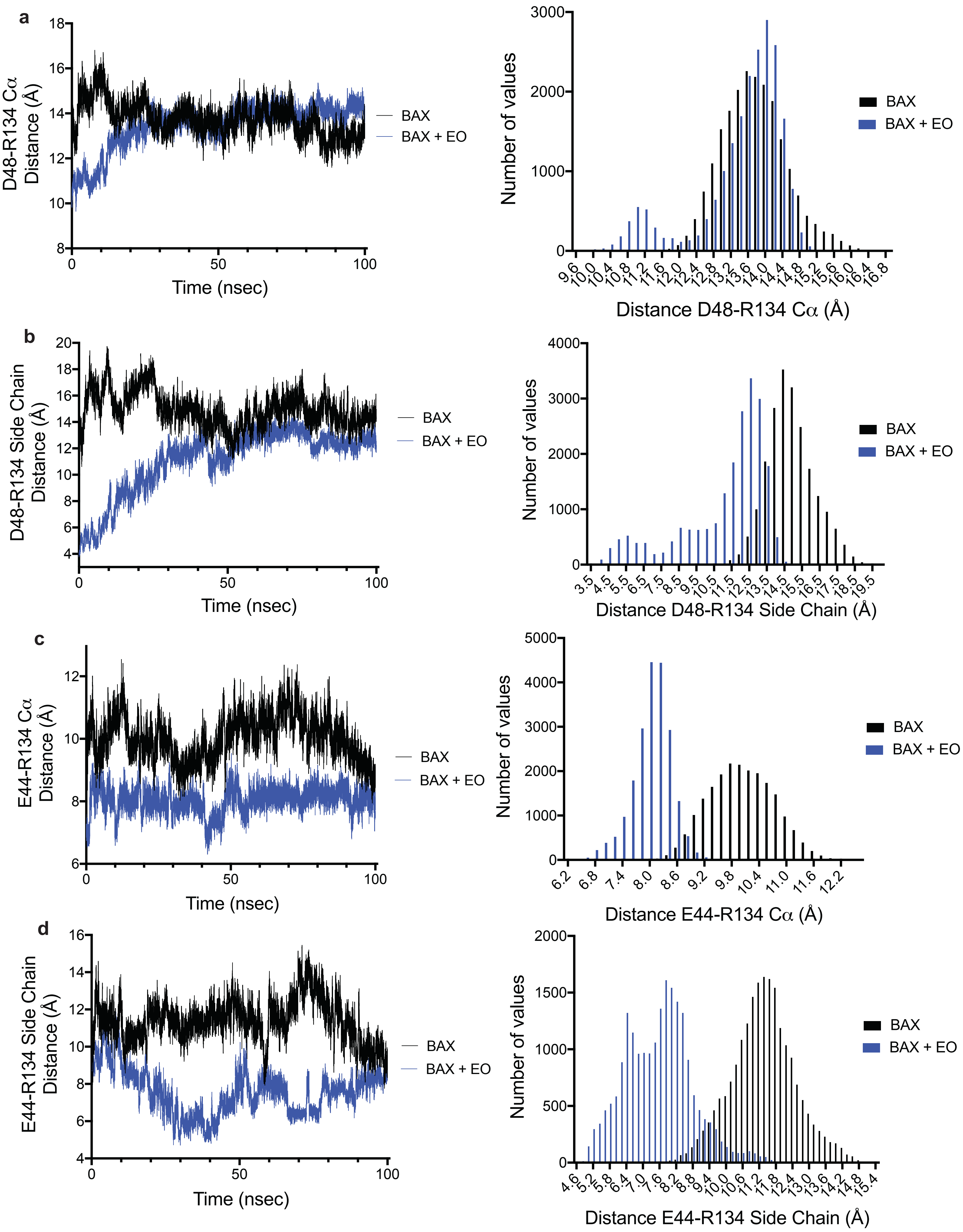
a
D48-R134 Cα Distance (Å)
Time (nsec)
BAX
BAX + EO
Number of values
Distance D48-R134 Cα (Å)
b
D48-R134 Side Chain Distance (Å)
Time (nsec)
BAX
BAX + EO
Number of values
Distance D48-R134 Side Chain (Å)
c
E44-R134 Cα Distance (Å)
Time (nsec)
BAX
BAX + EO
Number of values
Distance E44-R134 Cα (Å)
d
E44-R134 Side Chain Distance (Å)
Time (nsec)
BAX
BAX + EO
Number of values
Distance E44-R134 Side Chain (Å)

Supplementary Figure 7. Distance analysis of unbound BAX and BAX-Eltrombopag (EO) MD simulations: α1-α2 loop. a-**d**, Molecular dynamic simulation distances between the basic R134 and acidic D48 (**a**, **b**) and E44 (**c**, **d**) on α1- α2 loop of BAX. Distances are calculated from α-carbons (**a**, **c**) and side chain terminal carbons (**b**, **d**). Distances are represented as both distance with respect to time (left) and distance frequency histogram (right). All data represent mean n=3 simulations for both BAX and BAX-EO MD simulations. Source data has been provided.

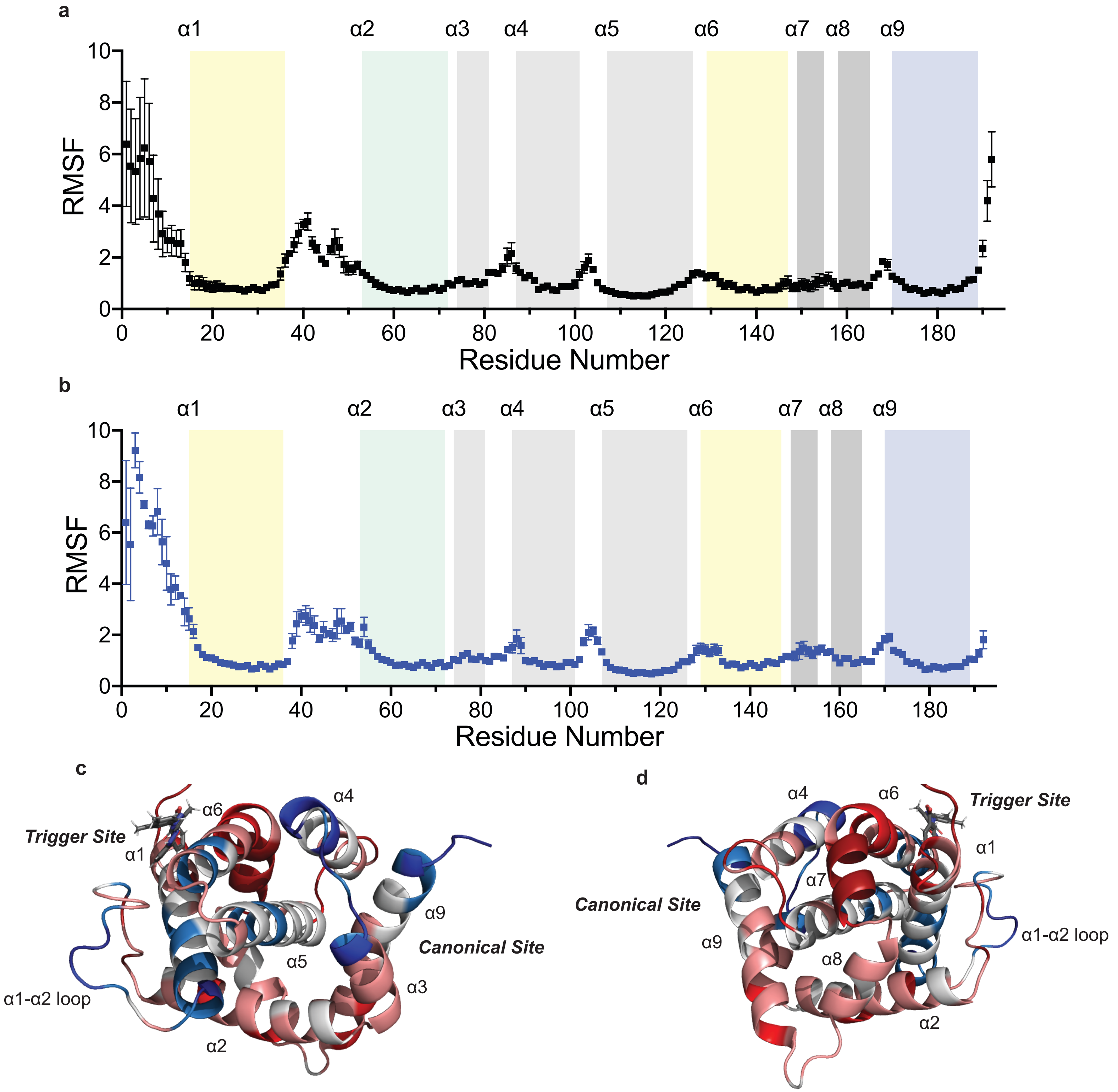

a
α1
α2
α3
α4
α5
α6
α7
α8
α9
RMSF
Residue Number
b
α1
α2
α3
α4
α5
α6
α7
α8
α9
RMSF
Residue Number
c
Trigger Site
α1
α6
α4
α9
Canonical Site
α5
α3
α1-α2 loop
α2
d
α4
α6
Trigger Site
α1
α7
Canonical Site
α9
α8
α1-α2 loop
α2

Supplementary Figure 8. Comparative analysis of molecular dynamics simulations. **a**-**b,** Root mean square fluctuation (RMSF) is plotted with respect to BAX residue number for BAX (**a**) and BAX-EO (**b**) molecular dynamic (MD) simulations. Data represent mean n=3 ± SEM for both BAX and BAX-EO simulations. **c**-**d,** Ribbon representation of BAX-EO complex with residues demonstrating increased and decreased RMSF highlighted in shades red and blue, respectively, corresponding to Fig. 4f. The BAX-EO complex is oriented to highlight areas of clustered decrease (**c**) and increase (**d**) in RMSF. Labels for helices and regions of interest (canonical site, trigger site) are included for clarity. Source data has been provided.

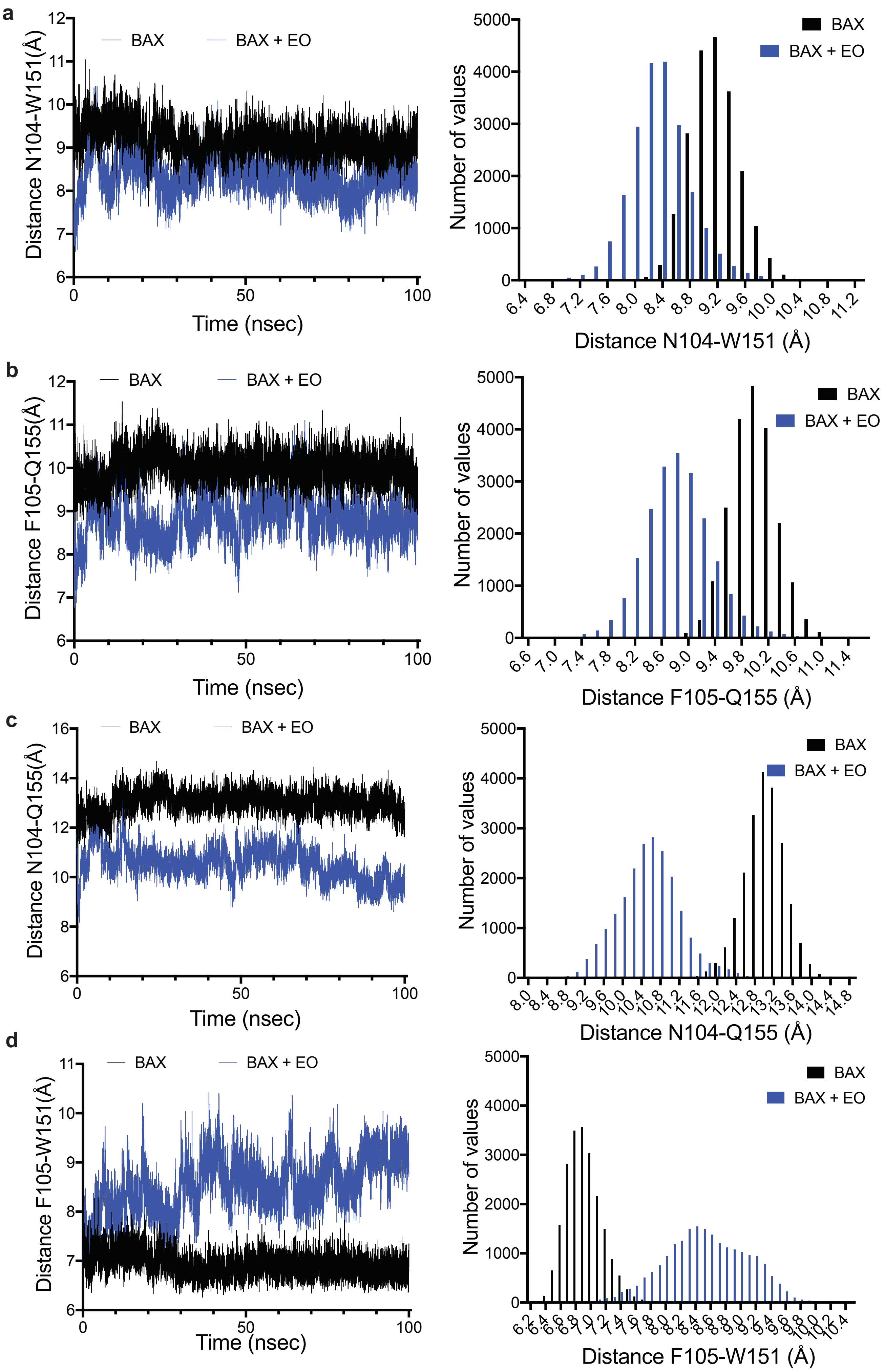
a
BAX
BAX + EO
Distance N104-W151(Å)
Time (nsec)
Number of values
Distance N104-W151 (Å)
b
BAX
BAX + EO
Distance F105-Q155(Å)
Time (nsec)
Number of values
Distance F105-Q155 (Å)
c
BAX
BAX + EO
Distance N104-Q155(Å)
Time (nsec)
Number of values
Distance N104-Q155 (Å)
d
BAX
BAX + EO
Distance F105-W151(Å)
Time (nsec)
Number of values
Distance F105-W151 (Å)

Supplementary Figure 9. Distance analysis of unbound BAX and BAX-Eltrombopag (EO) molecular dynamics simulations: α7/α4-α5 loop interface. a-d, Molecular dynamic simulation distances between α7 and α4-α5 loop residues N104-W151 (**a**), F105-Q155 (**b**), N104-Q155 (**c**), and F105-W151 (**d**). All distances are calculated from α-carbons. Distances are represented as both distance with respect to time (left) and distance frequency histogram (right). All data represent mean n=3 simulations for both BAX and BAX-EO MD simulations. Source data has been provided.

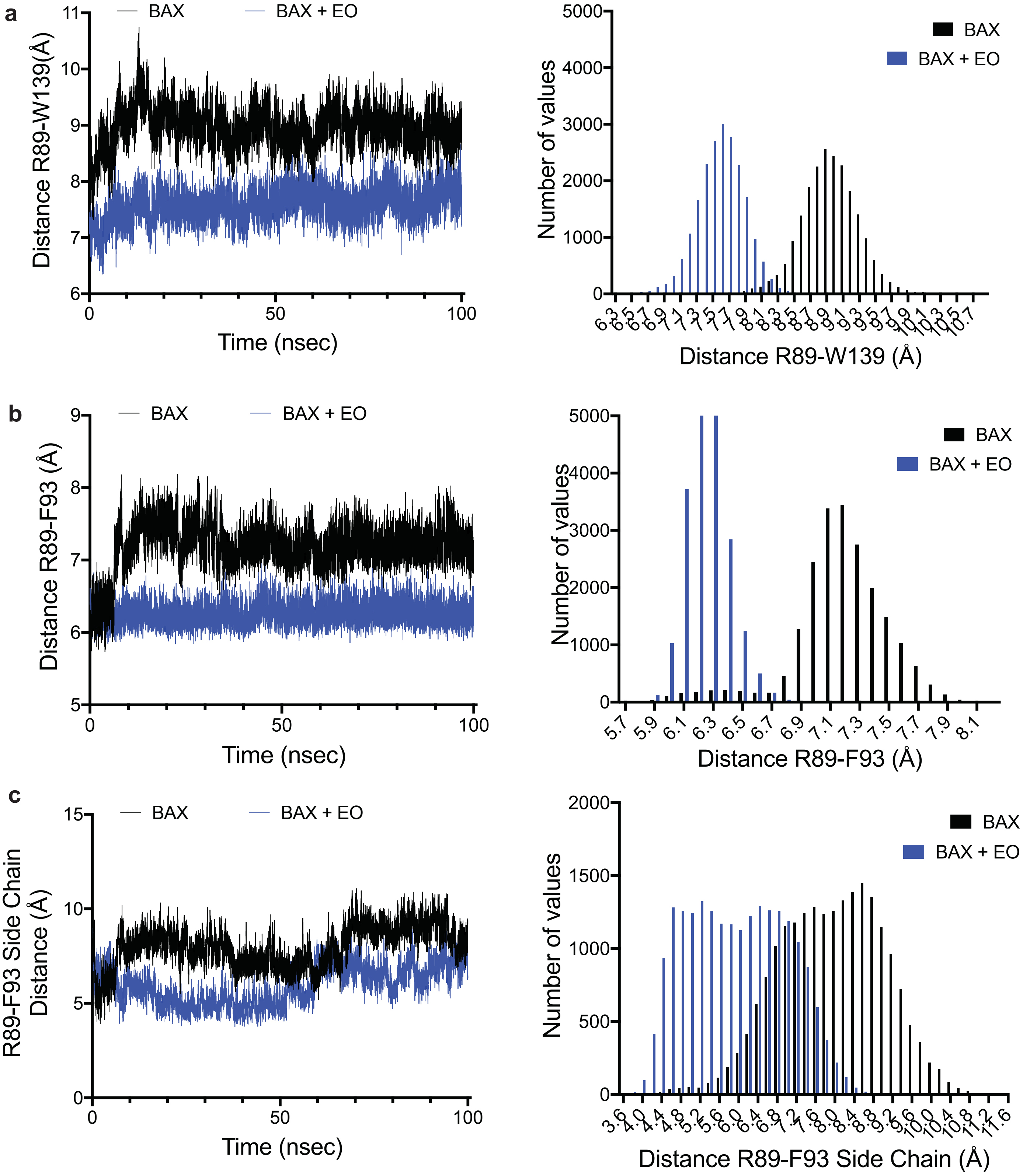

a
BAX
BAX + EO
Distance R89-W139(Å)
Time (nsec)
Number of values
Distance R89-W139 (Å)
b
BAX
BAX + EO
Distance R89-F93 (Å)
Time (nsec)
Number of values
5.7
5.9
6.1
6.3
6.5
6.7
6.9
7.1
7.3
7.5
7.7
7.9
8.1
Distance R89-F93 (Å)
c
BAX
BAX + EO
R89-F93 Side Chain
Distance (Å)
Time (nsec)
Number of values
Distance R89-F93 Side Chain (Å)

Supplementary Figure 10. Distance analysis of unbound BAX and BAX-Eltrombopag (EO) molecular dynamics simulations: α4-α6 interface. a-c, Molecular dynamic simulation distance between α4 and α6 residues R89-W139 (**a**), and R89-F93 (**b**). Distances is calculated from α-carbons. Distance between R89-F93 terminal carbons (**c**). Distance is represented as both distance with respect to time (left) and distance frequency histogram (right). Data represent mean n=3 simulations for both BAX and BAX-EO MD simulations. Source data has been provided.

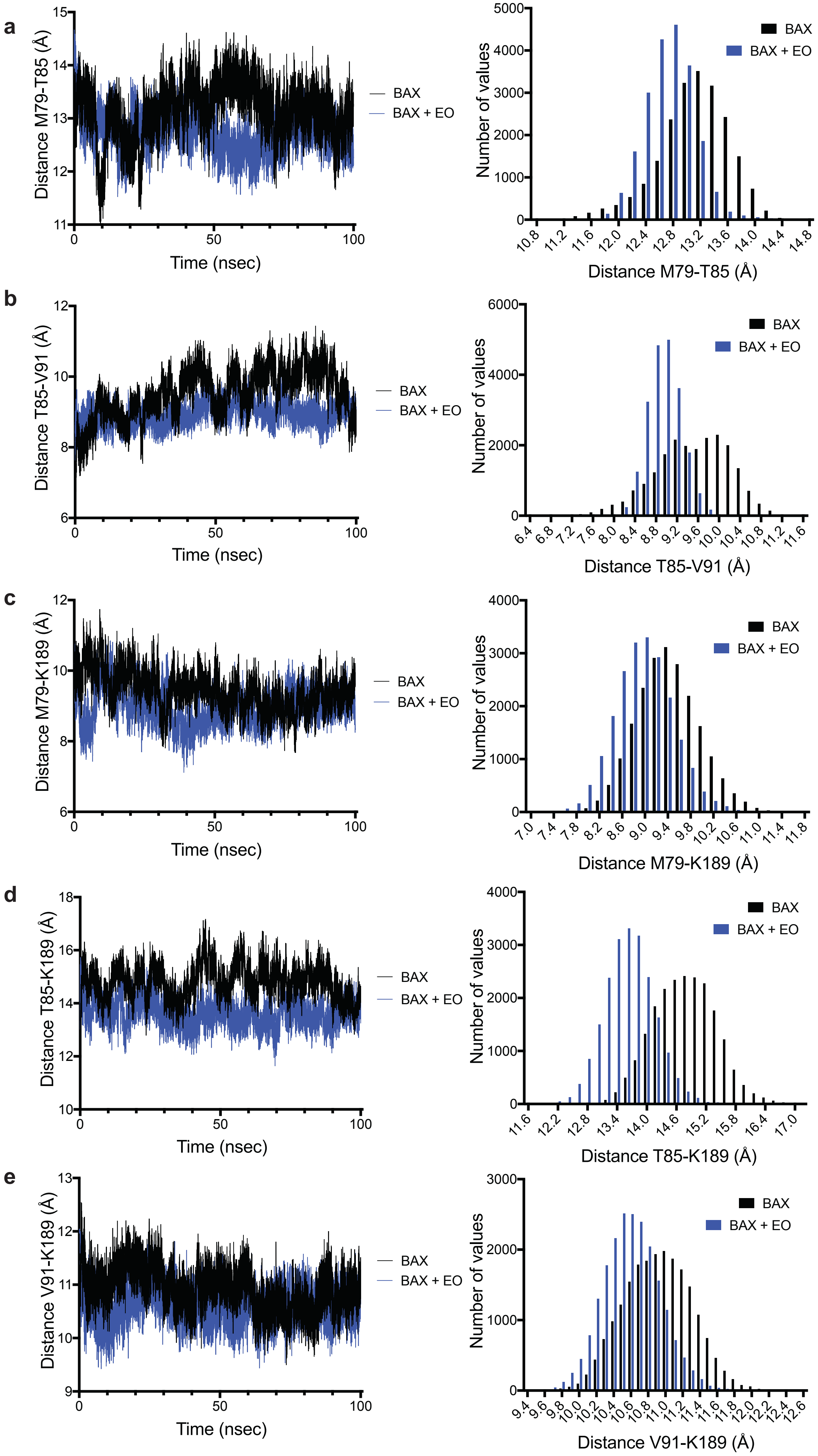

a
Distance M79-T85 (Å)
Time (nsec)
BAX
BAX + EO
Number of values
Distance M79-T85 (Å)
b
Distance T85-V91 (Å)
Time (nsec)
BAX
BAX + EO
Number of values
Distance T85-V91 (Å)
c
Distance M79-K189 (Å)
Time (nsec)
BAX
BAX + EO
Number of values
Distance M79-K189 (Å)
d
Distance T85-K189 (Å)
Time (nsec)
BAX
BAX + EO
Number of values
Distance T85-K189 (Å)
e
Distance V91-K189 (Å)
Time (nsec)
BAX
BAX + EO
Number of values
Distance V91-K189 (Å)

Supplementary Figure 11. Distance analysis of unbound BAX and BAX-Eltrombopag (EO) molecular dynamics simulations: α9/α3-α4 loop interface. a-e, Molecular dynamic simulation distances between canonical site residues M79-T85 (**a**), T85-V91 (**b**), M79-K189 (**c**) T85-K189 (**d**), V91-K189 (**e**). All distances are calculated from α-carbons. Distances are represented as both distance with respect to time (left) and distance frequency histogram (right). All data represent mean n=3 simulations for both BAX and BAX-EO MD simulations. Source data has been provided.

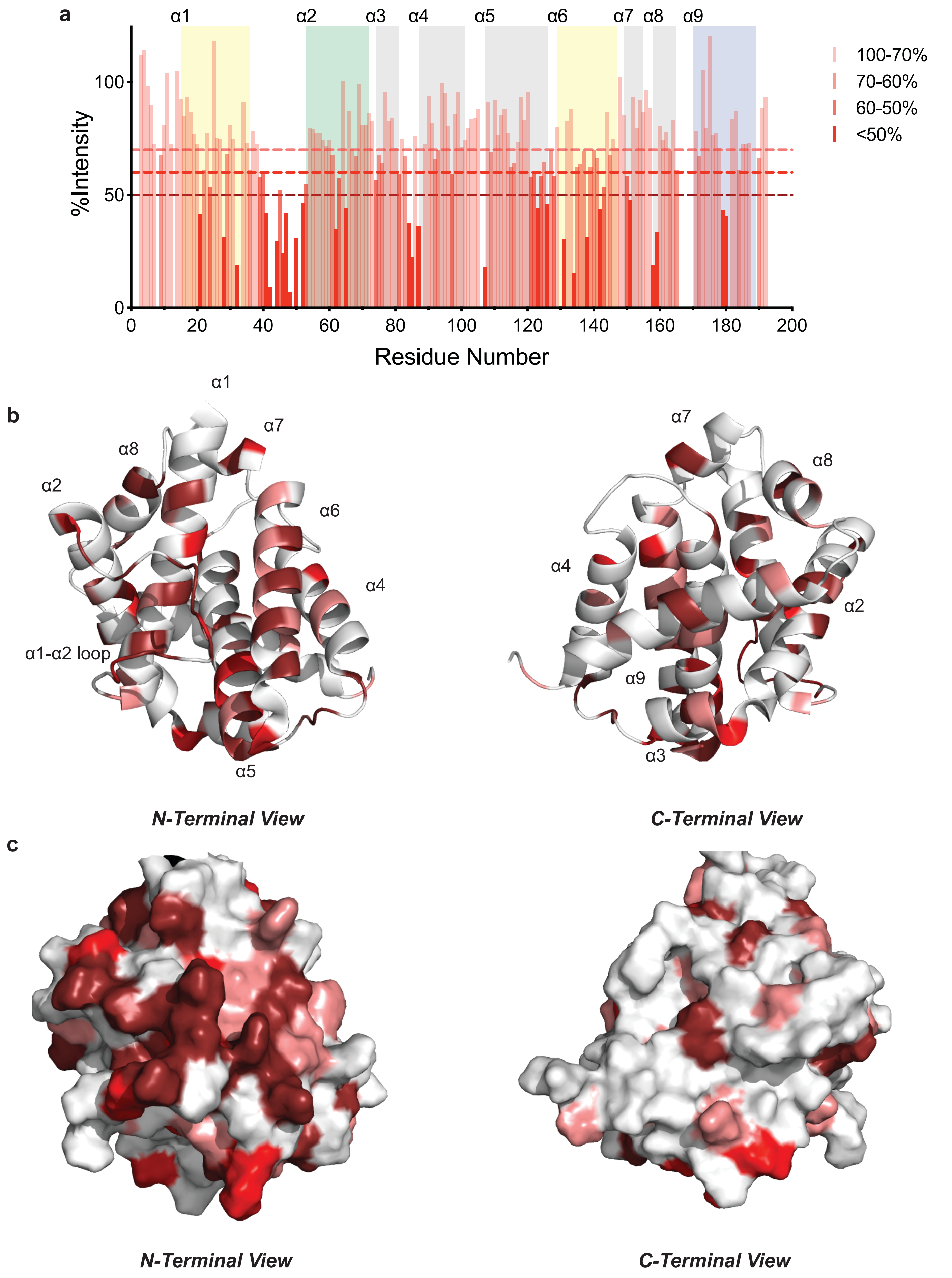
a
α1
α2
α3
α4
α5
α6
α7
α8
α9
%Intensity
0
50
100
0
20
40
60
80
100
120
140
160
180
200
Residue Number
100-70%
70-60%
60-50%
<50%
b
α1
α7
α8
α2
α6
α4
α1-α2 loop
α5
α7
α8
α4
α2
α9
α3
N-Terminal View
C-Terminal View
c
N-Terminal View
C-Terminal View

Supplementary Figure 12. Analysis of paramagnetic relaxation effects (PRE) on the BAX structure by PRE probe, hydroxy-TEMPO. **a,** Percentage intensity paramagnetic relaxation effect (PRE) of ^{15}N-labeled BAX in the presence of the soluble paramagnetic reagent hydroxy-TEMPO plotted with respect to BAX residue number. Intensities below the labeled threshold values are indicated in shades of red. Residues associated with the N-terminal trigger site, BH3-domain, canonical site, and transmembrane domain are highlighted in yellow, green, grey, and blue, respectively. **b**-**c,** Ribbon (**b**) and surface (**c**) representations of BAX with residues exhibiting significant PRE-highlighted in shades of red corresponding to (**a**). Helices are labeled and N-terminal (left) and C-terminal (right) views are provided for clarity. Source data has been provided.

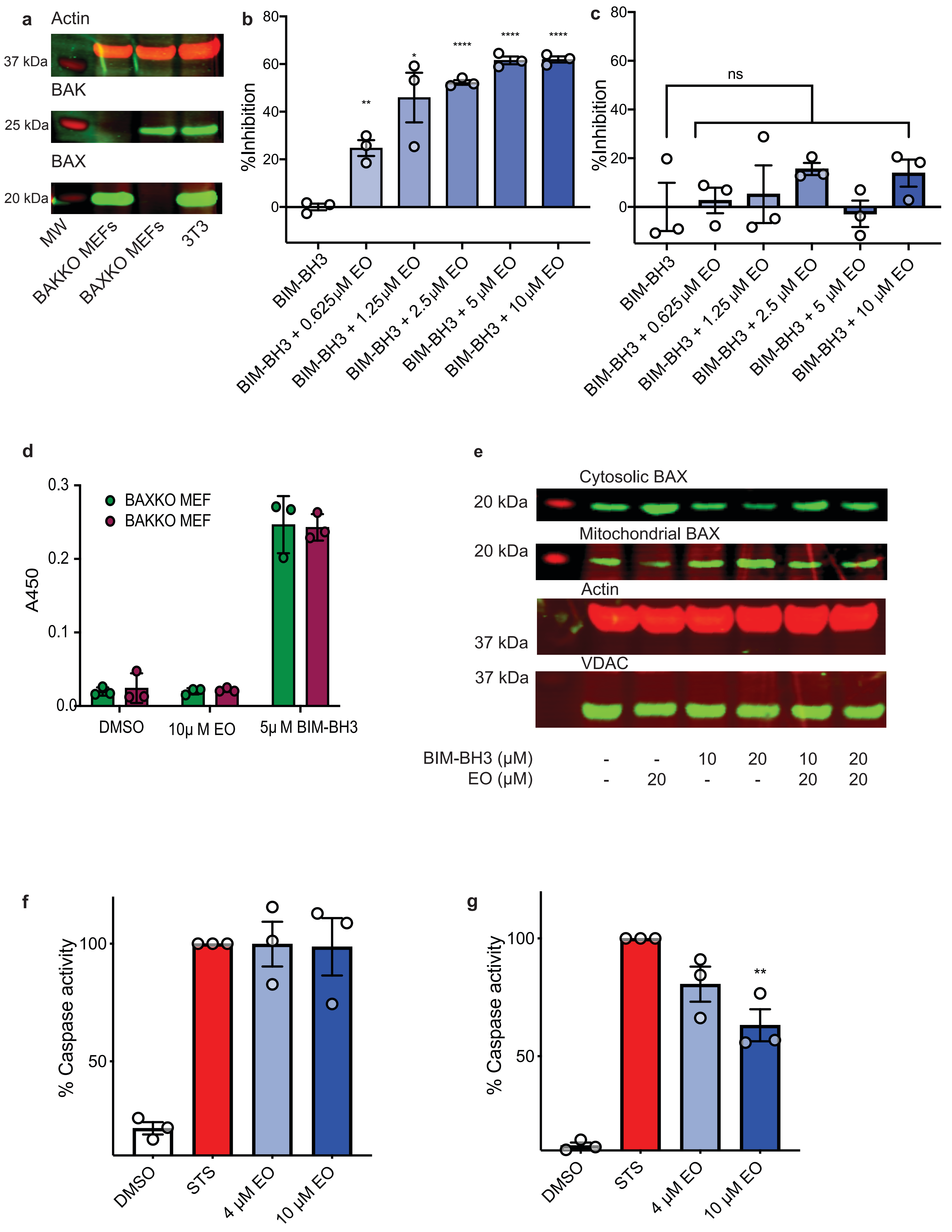

a
Actin
37 kDa
BAK
25 kDa
BAX
20 kDa
MW
BAKKO MEFs
BAXKO MEFs
3T3
b
%Inhibition
BIM-BH3
BIM-BH3 + 0.625μM EO
BIM-BH3 + 1.25μM EO
BIM-BH3 + 2.5μM EO
BIM-BH3 + 5 μM EO
BIM-BH3 + 10μM EO
c
ns
%Inhibition
BIM-BH3
BIM-BH3 + 0.625 μM EO
BIM-BH3 + 1.25 μM EO
BIM-BH3 + 2.5 μM EO
BIM-BH3 + 5 μM EO
BIM-BH3 + 10 μM EO
d
BAXKO MEF
BAKKO MEF
A450
DMSO
10μ M EO
5μ M BIM-BH3
e
Cytosolic BAX
20 kDa
Mitochondrial BAX
20 kDa
Actin
37 kDa
VDAC
37 kDa
BIM-BH3 (μM) - - 10 20 10 20
EO (μM) - 20 - - 20 20
f
% Caspase activity
DMSO
STS
4 μM EO
10 μM EO
g
% Caspase activity
DMSO
STS
4 μM EO
10 μM EO

Supplementary Figure 13. Eltrombopag inhibits BAX translocation and BAX-mediated apoptosis in cells. a, Western blot analysis of BAK KO MEFs, BAX KO MEFs, and 3T3 cells confirming the expression and knock-out of BAX or BAK. Data representative of n=3 independent cell lysate isolates. **b-c,** Percentage inhibition of BIM-BH3 induced mitochondrial cytochrome *c* release in $BAK^{-/-}$ mouse embryonic fibroblasts (MEFs) (**b**) and $BAX^{-/-}$ MEFs(**c**). **d,** A450 Absorbance corresponding to BIM-BH3 induced mitochondrial cytochrome c release for $BAX^{-/-}$ (green) and $BAK^{-/-}$ (maroon) MEFs demonstrates similar BIM-BH3 effects in either cell line. All data represent n=3 ± SEM and are representative of three independent experiments. **e**, Representative immunoblot analysis of BAX translocation in BAK KO MEF cells in response to BIM BH3 titration in the presence of 10 or 20 µM EO. Actin and VDAC are loading controls for cytosolic and mitochondrial fractions, respectively. **f-g,** Caspase 3/7 assay of $BAX^{-/-}$ mouse embryonic fibroblasts (MEFs) (**f**) and $BAK^{-/-}$ MEFs (**g**) in response to 3 µM STS and the presence or absence of various doses of EO for 6 hr. Data represent mean of n=3 ± SEM and are representative of three independent experiments. Two-sided *t*-test, **** $P < 0.0001$; ***$P < 0.001$;**$P < 0.01$; *$P < 0.05$; ns, $P > 0.05$ ((Left to right P values were 0.0023, 0.0119, 0.000007, 0.000009, 0.000005 for (**b**) 0.0055 for (**g**)). Source data has been provided.

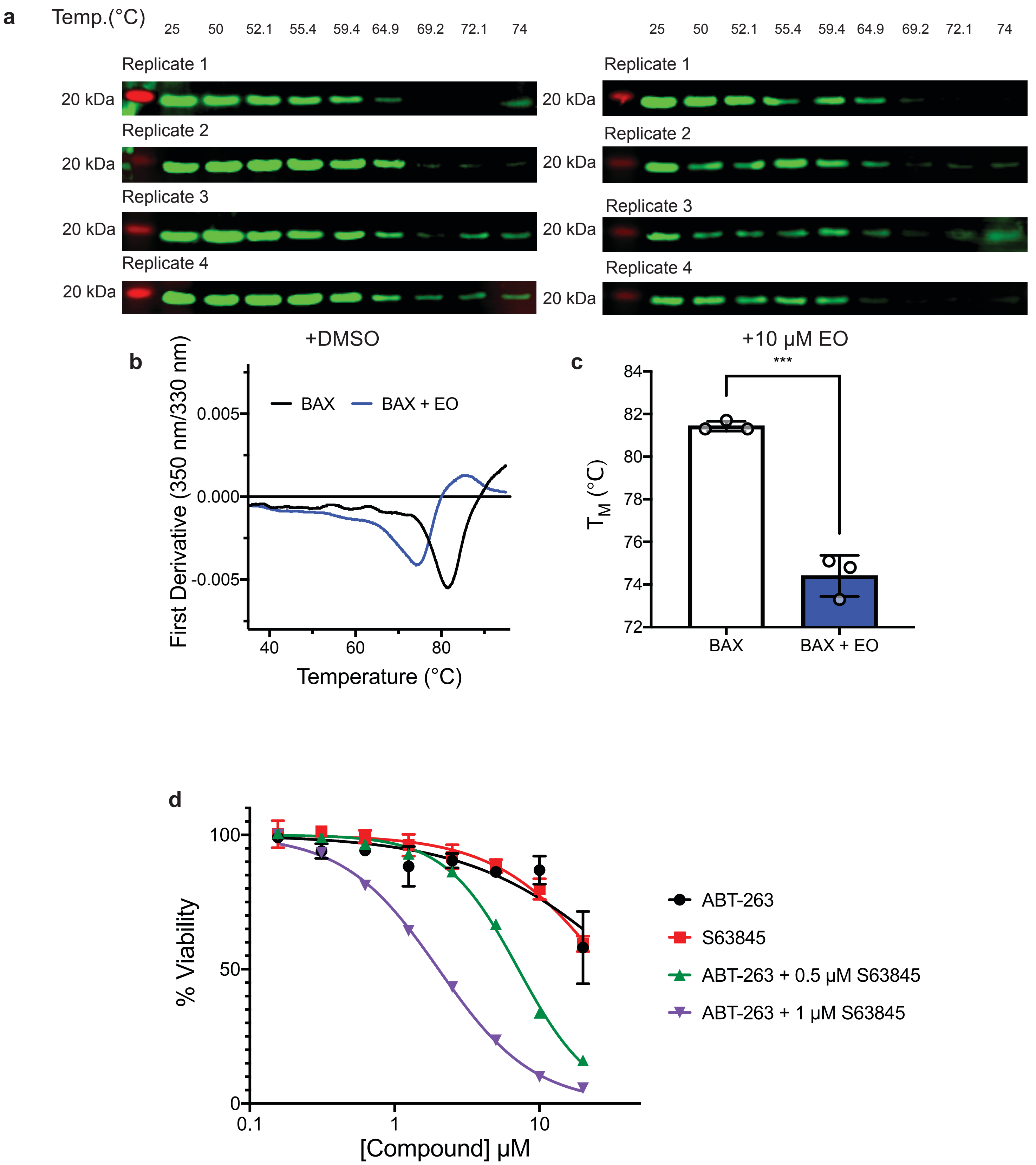
a
Temp.(°C)
25 50 52.1 55.4 59.4 64.9 69.2 72.1 74
Replicate 1
20 kDa
Replicate 2
20 kDa
Replicate 3
20 kDa
Replicate 4
20 kDa
+DMSO
25 50 52.1 55.4 59.4 64.9 69.2 72.1 74
Replicate 1
20 kDa
Replicate 2
20 kDa
Replicate 3
20 kDa
Replicate 4
20 kDa
+10 μM EO
b
BAX
BAX + EO
First Derivative (350 nm/330 nm)
0.005
0.000
-0.005
40
60
80
Temperature (°C)
c
***
84
82
80
78
76
74
72
T_M (°C)
BAX
BAX + EO
d
100
50
0
% Viability
0.1
1
10
[Compound] μM
ABT-263
S63845
ABT-263 + 0.5 μM S63845
ABT-263 + 1 μM S63845

Supplementary Figure 14. Eltrombopag engages and inhibits BAX in cells. a, Cellular thermal shift assay (CETSA) BAX melting curves for BAK KO MEFs treated with vehicle or 10 μM EO. Replicate western blots were quantified by fluorescence intensity using LiCor Odyssey Clx and normalized to generate melting curve in **Figure 6d**. Data representative of n=4 independent CETSA experiments with vehicle or 10 μM EO. **b,** Representative melting curve of recombinant BAX (black) and BAX + EO (blue) at a 1:10 ratio. Curves represent average of n=3 independent experiments. **c,** Calculated melting temperature (T_M) based on (**b**) of BAX (black) and BAX + EO (blue) at a 1:10 ratio. **d,** Percentage viability of 3T3 cells fibroblast cells treated with the indicated doses of ABT-263, S63845 as single agents or in combination. Data represent n=3 ± SEM of three independent experiments. Two-sided *t*-test, **** $P < 0.0001$; ***$P < 0.001$;**$P < 0.01$; *$P < 0.05$; ns, $P > 0.05$ (P value was 0.0003 for (**c**)). Source data has been provided.
