# Supplementary material for: Eltrombopag directly inhibits BAX and prevents cell death
Source: Nat Commun. 2021 Feb 18;12:1134. doi: 10.1038/s41467-021-21224-1 (PMC7892824; doi:10.1038/s41467-021-21224-1)
Supplement: Supplementary file 3 — Reporting Summary [file 41467_2021_21224_MOESM3_ESM.pdf]

nature research

Corresponding author(s): Evripidis Gavathiotis

Last updated by author(s): 2021-1-7

Reporting Summary

Nature Research wishes to improve the reproducibility of the work that we publish. This form provides structure for consistency and transparency in reporting. For further information on Nature Research policies, see our Editorial Policies and the Editorial Policy Checklist.

Statistics

For all statistical analyses, confirm that the following items are present in the figure legend, table legend, main text, or Methods section.

n/a	Confirmed	
☐	☒	The exact sample size (*n*) for each experimental group/condition, given as a discrete number and unit of measurement
☐	☒	A statement on whether measurements were taken from distinct samples or whether the same sample was measured repeatedly
☐	☒	The statistical test(s) used AND whether they are one- or two-sided<br>*Only common tests should be described solely by name; describe more complex techniques in the Methods section.*
☐	☒	A description of all covariates tested
☐	☒	A description of any assumptions or corrections, such as tests of normality and adjustment for multiple comparisons
☐	☒	A full description of the statistical parameters including central tendency (e.g. means) or other basic estimates (e.g. regression coefficient) AND variation (e.g. standard deviation) or associated estimates of uncertainty (e.g. confidence intervals)
☐	☒	For null hypothesis testing, the test statistic (e.g. F, t, r) with confidence intervals, effect sizes, degrees of freedom and P value noted<br>*Give P values as exact values whenever suitable.*
☒	☐	For Bayesian analysis, information on the choice of priors and Markov chain Monte Carlo settings
☒	☐	For hierarchical and complex designs, identification of the appropriate level for tests and full reporting of outcomes
☒	☐	Estimates of effect sizes (e.g. Cohen's d, Pearson's r), indicating how they were calculated

Our web collection on statistics for biologists contains articles on many of the points above.

Software and code

Policy information about availability of computer code

Data collection	Modeling data were collected and analyzed with Schrodinger software suite (Releases 2016-2019, Schrodinger, LLC) and Pymol (Version 2.3, The PyMOL Molecular Graphics System, Schrodinger, LLC). NMR data were collected and processed with Topspin software (Version 2.1, 2016, Bruker). MST Data was acquired NanoTemper MO.Control 2 (2019, NanoTemper).
Data analysis	Data analysis and statistical comparisons were performed by Graphpad Prisom 7.0-9.0 software. NMR data were analyzed with NMRView v.8.0.3. Structural and small molecule data were analyzed with modules SiteMap, GLIDE, EPIC, LIGPREP, MAESTRO, DESMOND, IFD, MM-GBSA using Schrodinger Software Suite version 2016-2019. Western blot data were analyzed with Image Studie 3.1. MST Data was analyzed using NanoTemper MO.Affinity Analysis 3 (2019, NanoTemper).

For manuscripts utilizing custom algorithms or software that are central to the research but not yet described in published literature, software must be made available to editors and reviewers. We strongly encourage code deposition in a community repository (e.g. GitHub). See the Nature Research guidelines for submitting code & software for further information.

Data

Policy information about availability of data

All manuscripts must include a data availability statement. This statement should provide the following information, where applicable:

- Accession codes, unique identifiers, or web links for publicly available datasets
- A list of figures that have associated raw data
- A description of any restrictions on data availability

All data generated or analyzed during this study are included in this published article and its supplementary information files and are available from the corresponding author on a reasonable request. Source data are provided with this paper. The following publicly available data sets were used in the production of this manuscript:

PDB 1F16 DOI: 10.2210/pdb1F16/pdb
PDB 2K7W DOI: 10.2210/pdb2K7W/pdb

Field-specific reporting

Please select the one below that is the best fit for your research. If you are not sure, read the appropriate sections before making your selection.

[x] Life sciences [ ] Behavioural & social sciences [ ] Ecological, evolutionary & environmental sciences

For a reference copy of the document with all sections, see nature.com/documents/nr-reporting-summary-flat.pdf

Life sciences study design

All studies must disclose on these points even when the disclosure is negative.

Sample size	Sample size were designed based on previous experience with assays on related projects (Garner et al. Nat. Chem. Bio. 2019 DOI: 10.1038/s41589-018-0223-0, Amgalan et al. Nat. Cancer 2020 DOI: 10.1038/s43013-020-0039-1) and variability of the response deviating from the mean as presented in the graphs and figure legends. Sample sizes and statistical data are reported in figure legends.
Data exclusions	No data were excluded from the analyses.
Replication	Experimental findings were replicated at least 2 or more times as indicated in the figure legends and replication was successful at all attempts.
Randomization	Allocation was random.
Blinding	Blinding was not required in this study as all comparisons were made using quantitative analysis of computational, biochemical, or cellular data with no animal or human subjects and randomized group allocation was not performed.

Reporting for specific materials, systems and methods

We require information from authors about some types of materials, experimental systems and methods used in many studies. Here, indicate whether each material, system or method listed is relevant to your study. If you are not sure if a list item applies to your research, read the appropriate section before selecting a response.

Materials & experimental systems

n/a	Involved in the study	
[ ]	[x]	Antibodies
[ ]	[x]	Eukaryotic cell lines
[x]	[ ]	Palaeontology and archaeology
[x]	[ ]	Animals and other organisms
[x]	[ ]	Human research participants
[x]	[ ]	Clinical data
[x]	[ ]	Dual use research of concern

Methods

n/a	Involved in the study	
[x]	[ ]	ChIP-seq
[x]	[ ]	Flow cytometry
[x]	[ ]	MRI-based neuroimaging

Antibodies

Antibodies used	6A7 mouse monoclonal antibody (Santa Cruz Cat. #sc-23959), 15 µL per reaction<br>BAX monoclonal antibody (Cell Signaling Cat. #2772) 1:1000 dilution for western blot, 1:100 dilution for imaging<br>anti-BAK (Millipore Cat. #06-536) 1:1000 dilution<br>anti-β-Actin (Sigma Cat. #A1978) 1:5000 dilution<br>anti-VDAC (Cell Signaling Cat. #4661) 1:1000 dilution<br>anti-TOMM20 (Sigma Cat. #ST1705) 1:100 dilution<br>IRdye800-conjugated goat anti-rabbit IgG (LI-COR Biosciences Cat. #925-32211) 1:5000 dilution<br>IRdye800-conjugated goat anti-mouse IgG (LI-COR Biosciences Cat. #925-32210) 1:5000 dilution<br>Goat anti-Rabbit IgG (H+L) Cross-Adsorbed Secondary Antibody, Alexa Fluor 488 (ThermoFisher Cat. #11008) 1:500 dilution<br>Goat anti-Mouse IgG (H+L) Highly Cross-Adsorbed Secondary Antibody, Alexa Fluor 546 (ThermoFisher Cat. #11030) 1:500 dilution
Validation	6A7 mouse monoclonal antibody was validated in HT-1080 , BJAB , Ramos, and MDA-MB-231 whole cell lysates by Santa Cruz. Sample PMID: 30049712<br>BAX monoclonal antibody was validated in extracts from HeLa cells by Cell Signaling. Sample PMID: 31899991<br>anti-BAK was validated in extracts from A431, C2C12, HEK293, and HepG2 among other cell and tissue samples by Millipore. Sample PMID: 26231047<br>anti-β-Actin was validated in extracts from HeLa, JURKAT, COS7, and NIH-3T3 among other cell samples by Sigma. Sample PMID: PMID 22109529

anti-VDAC was validated in extracts from MCF-7, Jurkat, HCI-H441, and Hek293 among other cell samples by Cell Signaling. Sample PMID: 32978498
anti-TOMM20 was validated in extracts from HeLa, PC-12, and NIH-3T3 cell samples by Millipore. Sample PMID: 27771514

Eukaryotic cell lines

Policy information about cell lines

Cell line source(s)	NIH-3T3 (reffered to as 3T3), BAK-/- Mouse Embryonic Fibroblasts (reffered to as BAKKO MEFs), and BAX -/- Mouse Embryonic Fibroblasts (reffered to as BAXKO MEFs) were purchased from ATCC.
Authentication	Cell lines were authenticated from their vendor. ATCC uses morphology, karyotyping, and PCR based approaches to confirm the identity of human and mouse cell lines and to rule out both intra- and interspecies contamination.
Mycoplasma contamination	All cell lines were tested negative for mycoplasma contamination.
Commonly misidentified lines (See ICLAC register)	No commonly misidentified lines were used in our study.
