# Supplementary material for: Eltrombopag directly inhibits BAX and prevents cell death
Source: Nat Commun. 2021 Feb 18;12:1134. doi: 10.1038/s41467-021-21224-1 (PMC7892824; doi:10.1038/s41467-021-21224-1)
Supplement: Supplementary file 2 — Peer Review [file 41467_2021_21224_MOESM2_ESM.pdf]

Editorial Note: This manuscript has been previously reviewed at another journal that is not operating a transparent peer review scheme. This document only contains reviewer comments and rebuttal letters for versions considered at *Nature Communications.*
